# *mlo*‐based powdery mildew resistance in hexaploid bread wheat generated by a non‐transgenic TILLING approach

**DOI:** 10.1111/pbi.12631

**Published:** 2016-09-25

**Authors:** Johanna Acevedo‐Garcia, David Spencer, Hannah Thieron, Anja Reinstädler, Kim Hammond‐Kosack, Andrew L. Phillips, Ralph Panstruga

**Affiliations:** ^1^ Unit of Plant Molecular Cell BiologyInstitute for Biology IRWTH Aachen UniversityAachenGermany; ^2^ Department of Plant Biology and Crop ScienceRothamsted ResearchWest CommonHarpendenHertfordshireAL5 2JQ, UK

**Keywords:** Targeting Induced Local Lesions in Genomes, powdery mildew, *Mlo*, hexaploid bread wheat, *Blumeria graminis*, plant disease resistance

## Abstract

Wheat is one of the most widely grown cereal crops in the world and is an important food grain source for humans. However, wheat yields can be reduced by many abiotic and biotic stress factors, including powdery mildew disease caused by *Blumeria graminis* f.sp. *tritici* (*Bgt*). Generating resistant varieties is thus a major effort in plant breeding. Here, we took advantage of the non‐transgenic Targeting Induced Lesions IN Genomes (TILLING) technology to select partial loss‐of‐function alleles of *TaMlo*, the orthologue of the barley *Mlo* (*Mildew resistance locus o*) gene. Natural and induced loss‐of‐function alleles (*mlo*) of barley *Mlo* are known to confer durable broad‐spectrum powdery mildew resistance, typically at the expense of pleiotropic phenotypes such as premature leaf senescence. We identified 16 missense mutations in the three wheat *TaMlo* homoeologues, *TaMlo*‐*A1*,* TaMlo*‐*B1* and *TaMlo*‐*D1* that each lead to single amino acid exchanges. Using transient gene expression assays in barley single cells, we functionally analysed the different missense mutants and identified the most promising candidates affecting powdery mildew susceptibility. By stacking of selected mutant alleles we generated four independent lines with non‐conservative mutations in each of the three *TaMlo* homoeologues. Homozygous triple mutant lines and surprisingly also some of the homozygous double mutant lines showed enhanced, yet incomplete, *Bgt* resistance without the occurrence of discernible pleiotropic phenotypes. These lines thus represent an important step towards the production of commercial non‐transgenic, powdery mildew‐resistant bread wheat varieties.

## Introduction

Bread wheat (*Triticum aestivum*) is the third largest cultivated crop after maize and rice and the second in terms of dietary intakes. By 2013, global wheat grain production reached 712 million tonnes (http://faostat.fao.org), and by 2050 an estimated increase of a further 60% will be required to meet the demands of our growing population (http://wheatinitiative.org).

Allohexaploid wheat has a genome of 17 Gb in size, of which more than 80% is composed of repetitive transposable elements. Genetically, it has the structure of three independent genomes in one species (*AABBDD* genome), since meiotic pairing of homoeologous chromosomes is prevented through the action of *Ph* (*pairing homoeologous*) genes (IWGSC, 2014).

Yield in wheat can be reduced by abiotic factors, pests or diseases caused by pathogens. A global disease threat is powdery mildew caused by *Blumeria graminis* f.sp. *tritici* (*Bgt*) (Conner *et al*., 2003; Dean *et al*., 2012), an obligate biotrophic fungus (ascomycete) of the order Erysiphales (Glawe, 2008). However, more recently, *B. graminis* f.sp. *triticale* has also been reported to reproduce on wheat (Menardo *et al*., 2016). Control of the powdery mildew disease caused by *Bgt* in wheat is performed mainly by using fungicides and varieties containing *R* (resistance) genes, the latter typically conferring isolate‐specific protection (Dean *et al*., 2012). Going forward, the most cost effective solution to control this disease is by exploiting genetic resources to provide enhanced, durable resistance.

In barley, natural and induced loss‐of‐function mutations of the *Mildew resistance locus o* (*Mlo*) gene confer broad‐spectrum resistance against most *B. graminis* f.sp. *hordei* (*Bgh*) isolates, an effect that has been shown to last in the field for more than 30 years (Jørgensen, 1992; Lyngkjær *et al*., 2000). *Mlo* is a member of an ancient eukaryotic gene family that is conserved throughout the plant kingdom (Kusch *et al*., 2016), and although its role in powdery mildew resistance has been well studied in various species, the biochemical function of Mlo proteins is still unknown (Acevedo‐Garcia *et al*., 2014).

On susceptible host plants, once sporelings of the pathogen land on the leaf or stem surface, these germinate and form an appressorium within two hours. The appressorium attempts to penetrate the epidermal cell layer by generating a penetration peg. If the pathogen successfully enters the host cell, in the following hours of infection the penetration peg enlarges to develop the feeding structure known as haustorium. Thereafter, the pathogen will complete its asexual life cycle on the leaf surface with the development of epiphytic hyphae, the production of conidiophores and the release of new spores (reviewed in Glawe, 2008). In the case of resistant *mlo* plants, a near‐complete arrest of pathogen growth occurs at the penetration stage where the germinating spore is not able to develop a haustorium (Jørgensen and Mortensen, 1977). However, in barley *mlo* lines resistance is typically associated with pleiotropic phenotypes such as the spontaneous deposition of callose‐containing cell wall appositions, early chlorophyll decay and spontaneous mesophyll cell death, which together lead to chlorotic and necrotic leaf flecking and have been interpreted as signs of premature leaf senescence (Peterhänsel *et al*., 1997; Piffanelli *et al*., 2002; Schwarzbach, 1976; Wolter *et al*., 1993).

In wheat, the three orthologues of barley *Mlo*,* TaMlo‐A1*,* ‐B1* and ‐*D1* (Konishi and Sasanuma, 2010), are located on chromosomes 5AL, 4BL and 4DL (Elliott *et al*., 2002). In contrast to barley, occurrence of natural wheat *mlo* mutants has not been reported. This is likely due to its hexaploid nature, which may require mutation of all six gene copies to generate a resistance phenotype that would be detectable within a breeding programme. Previously, Elliott and co‐workers showed that one of the wheat orthologues of barley *Mlo*,* TaMlo‐B1*, can complement powdery mildew‐resistant barley *mlo* mutants at the single‐cell level (Elliott *et al*., 2002). Furthermore, Várallyay and colleagues used virus‐induced gene silencing (VIGS) to demonstrate that RNAi silencing of the *TaMlo* homoeologues in wheat results in powdery mildew resistance (Várallyay *et al*., 2012). Most recently, the teams of Caixia Gao and Jin‐Long Qiu took advantage of the TALEN (transcription activator‐like effector nuclease) genome editing technology to generate transgenic winter wheat plants containing simultaneous knockout lesions in the three *TaMlo* homoeologues. Compared to the respective parental line, these plants were fully resistant against *Bgt* infection (Wang *et al*., 2014).

Targeting Induced Local Lesions IN Genomes (TILLING), first introduced 16 years ago by McCallum and collaborators (McCallum *et al*., 2000), is a powerful approach that integrates chemical mutagenesis with a high‐throughput detection method to identify single‐nucleotide mutations in a specific region of a gene of interest. In principle, TILLING can be used in different plant species independent of their ploidy. Interestingly, polyploid species can tolerate higher mutation densities than diploids (Slade *et al*., 2005; Uauy *et al*., 2009; Wang *et al*., 2012). This feature translates into a far smaller population size that needs to be screened to reach saturated mutagenesis in a polyploid species (Kurowska *et al*., 2011). Chemical mutagenesis combined with TILLING provides an ample spectrum of mutations, where a pool of allelic variations can result in a range of weak to strong phenotypes (Slade *et al*., 2005). To date there are several TILLING resources available for various crop species such as hexaploid and durum wheat, barley, rice, tomato, maize, sorghum, soybean and potato (reviewed in Chen *et al*., 2014b). Although until recently no TILLING‐derived crop variety has been released commercially, they represent a great advantage for plant breeding (especially in Europe) since these varieties will be considered non‐transgenic (Chen *et al*., 2014b).

In wheat (diploid, tetraploid or hexaploid), several genes involved in starch synthesis have been targeted by TILLING, for example, *Waxy* (Rawat *et al*., 2012; Slade *et al*., 2005), *Starch Branching Enzyme II* (*SBEII*) (Botticella *et al*., 2011; Sestili *et al*., 2015; Slade *et al*., 2012; Uauy *et al*., 2009) and *Starch Synthase II* (*Sgp‐1*/*SSII* (Dong *et al*., 2009; Sestili *et al*., 2009). Additionally, genes involved in other processes such as carotenoid content (Colasuonno *et al*., 2016), grain width (Simmonds *et al*., 2016), flowering (Chen *et al*., 2014a) or vernalization (Chen and Dubcovsky, 2012), have been also targeted by TILLING.

Currently, wheat breeders have a great challenge to select for varieties that on the one hand have a minimal disturbance in growth, development and fertility, thereby maintaining good grain quality and enhancing grain yield, and on the other hand exhibit improved resistance to pests and pathogens. At least in Europe, the deployment of transgenic plants (including those resulting from genome editing approaches) is still socially and politically contentious in plant breeding, agriculture as well as the food, feed and drinks industries. Therefore, methods that are indisputably non‐transgenic are favoured for the development of new varieties.

In this study, we generated hexaploid bread wheat lines with enhanced resistance to the common powdery mildew disease. Our approach made use of the non‐transgenic TILLING technology to select four different lines containing missense mutations in the *TaMlo‐A1*,* TaMlo‐B1* and *TaMlo‐D1* homoeologues. Triple and some double mutant lines showed enhanced powdery mildew resistance compared to respective wild‐type (WT) plants. So far these mutant plants did not show discernible abnormalities in growth and development.

## Results

### Cloning of genomic sequences of the wheat *TaMlo‐A1*,* TaMlo‐B1* and *TaMlo‐D1* homoeologues

**Figure 1:**
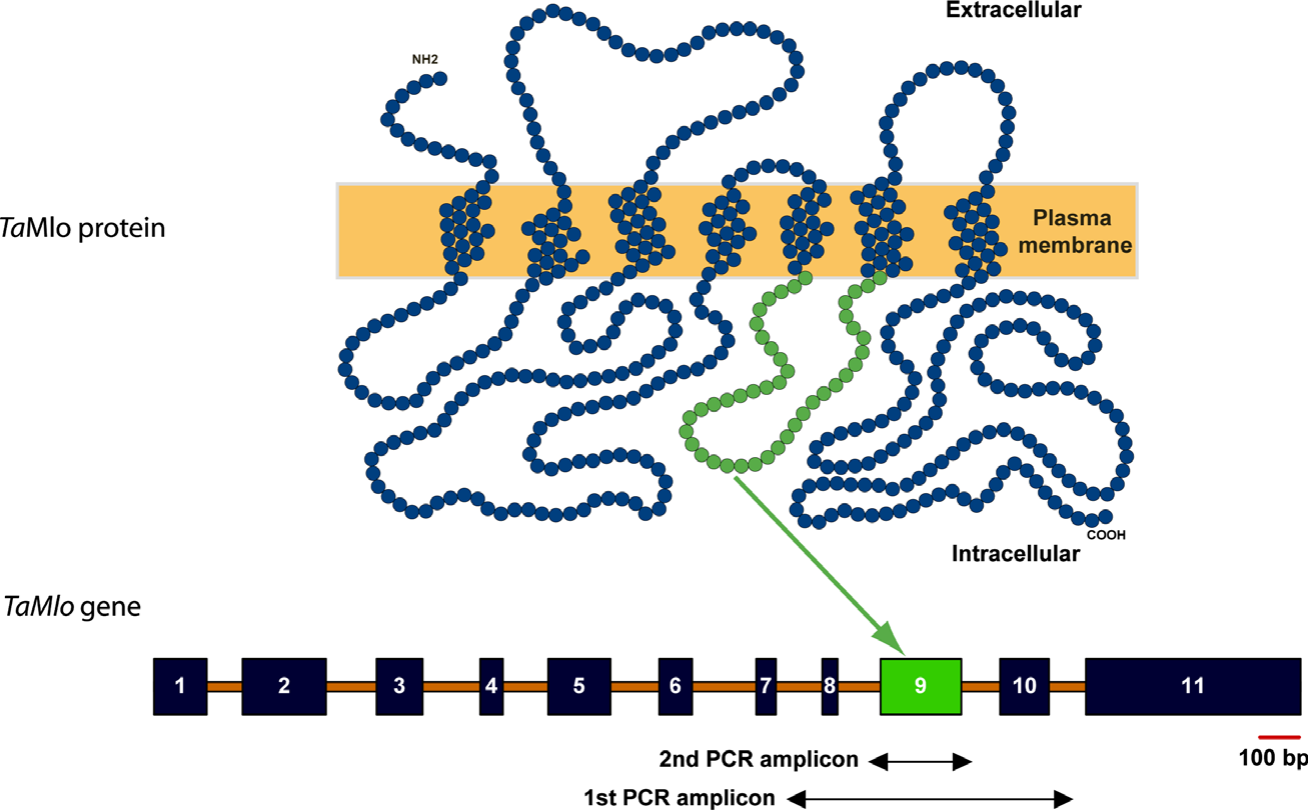
Exon 9 of *TaMlo* is the target for the TILLING screen. The scheme illustrates the predicted topology of the 7 transmembrane domain *Ta*Mlo proteins (top) and the experimentally determined common exon‐intron structure of the corresponding *TaMlo* homoeologues (bottom). The 11 exons are indicated by dark boxes, introns by orange lines. The third cytoplasmic loop of *Ta*Mlo, depicted in green and encoded by exon 9, was the target for TILLING. Arrows below the *TaMlo* gene model specify the amplicons of the 1st and 2nd round PCR. A scale bar, shown in red, is given below exon 11 (100 bp).

An essential requirement to perform a TILLING screening is the development of primers for gene‐specific polymerase chain reaction (PCR) amplification. A limiting factor in hexaploid wheat in this respect is the very high nucleotide similarity between the coding sequences of its homoeologous genes, which often constrains primer design. This is also the case for *TaMlo*, which exhibits 95%, 96% and 97% nucleotide sequence identity between the coding sequences of the A and B, A and D, and B and D genomes, respectively (File S1). To overcome this limitation, homoeologue‐specific PCR primers are frequently designed on the basis of intron sequences, which typically show a higher degree of nucleotide sequence polymorphisms than the exonic coding sequences. We thus first opted to obtain genomic DNA sequence information for the three *TaMlo* homoeologues. Based on the available known cDNA sequences in NCBI: (i) *TaMlo‐A1*: AF361933 and AX063298; (ii) *TaMlo‐B1*: AF361932, AX063294, AF384145; and (iii) *TaMlo‐D1*: AX063296, we designed common oligonucleotide primers, predicted to equally bind to all three *TaMlo* homoeologues, and used them to amplify and clone several overlapping genomic *TaMlo* fragments from DNA obtained from the hexaploid spring wheat cultivar (cv.) Cadenza. Following *in silico* assembly of the amplicons and also taking information from the known cDNA sequences into account, we obtained almost complete genomic assemblies representing the three *TaMlo* homoeologues. These were reconciled with the manually annotated sequences generated on the basis of the available wheat genome survey data to finally produce tentative genomic *TaMlo* consensus sequences (cv. Cadenza and cv. Chinese Spring hybrid sequences; File S2). The determined exon/intron structure of the *TaMlo* homoeologues is similar to the one of barley *Mlo* (Büschges *et al*., 1997) and comprises 11 exons each (Figure 1). The deduced *Ta*Mlo protein sequences are each one amino acid longer (534 amino acids) than barley Mlo (533 amino acids); consequently, as a result of small gaps in the alignment between wheat and barley Mlo proteins, the amino acid numbering after position 115 is shifted by +1 in the former compared to the latter.

### TILLING target and primer design

Previously, we identified the second and third cytoplasmic loop of the barley Mlo protein as relevant regions for its powdery mildew susceptibility‐conferring activity. Mutations in these parts of the Mlo protein that lead to a single amino acid exchange (missense mutations) often result in a loss‐of‐function of the protein and thus resistance against the powdery mildew pathogen (Reinstädler *et al*., 2010). Hence, we selected the third cytoplasmic loop, encoded by exon 9 of the *TaMlo* homoeologues, as the target region for the TILLING screening in wheat (Figure 1). We developed homoeologue‐specific primers for a 1st round PCR to amplify a product (between 750 and 1700 bp in size, depending on the *TaMlo* homoeologue) that contains the target exon (Figure 1 and Table S1). In addition, as High‐Resolution Melt (HRM) analysis is most accurate on DNA fragments up to ~300 bp in size (Reed and Wittwer, 2004), homoeologue‐specific primers were designed for a 2nd round PCR with a maximum amplicon size of 310 bp (Figure 1 and Table S1). Primer specificity was confirmed by amplification from genomic template DNA of cv. Cadenza followed by direct amplicon sequencing.

### Identification of *Tamlo* mutant candidates by TILLING

The ethyl methane sulfonate (EMS)‐mutagenized population of spring bread wheat cv. Cadenza has been previously described (Rakszegi *et al*., 2010). Genomic DNA samples derived from ~2020 M_2_ individuals were pooled twofold and employed as templates for 1st round PCR amplification in the target region using the validated homoeologue‐specific primer pairs. The primary PCR product was used as template for a 2nd round of PCR and the resulting ~300 bp amplicons were subjected to HRM analysis to detect mutations in the target exon of the *TaMlo* homoeologues.

After screening the entire population, a total of 76 candidate mutants were identified (28, 27 and 21 in *TaMlo‐A1*,* TaMlo‐B1* and *TaMlo‐D1*, respectively, Table 1). Twenty of these candidate mutations could not be confirmed in a secondary round of analysis; these were considered false positives and discarded from further study. Among the remaining 56 mutants, 14 mutations were located in intron regions flanking exon 9, but did not affect the consensus GT/AG splice junctions, and 26 were silent (synonymous mutations, Table 1), resulting in an unaltered *Ta*Mlo amino acid sequence. The remaining 16 mutants each represented missense mutations (non‐synonymous mutations, Table 2); nonsense mutations were not found in this screen. At the nucleotide level, the identified missense mutations were all transitions of the type C → T or G → A, as expected for EMS mutagenesis (Table 2). Furthermore, the mutations covered a broad spectrum of amino acid exchanges, ranging from substitutions of amino acids with similar biophysical properties (e.g. S315N, V323I, A354V) to exchanges with dramatic alterations in biophysical properties (e.g. R313W, P335L, G296E). Three of the mutations (G319R, A320T and T345M) were found in the *TaMlo‐B1* as well as in the *TaMlo‐D1* genome (Table 2). Interestingly, mutations G319R (*TaMlo‐B1* and *TaMlo‐D1*) and P335L (*TaMlo‐D1*) were previously identified as sequence variants with the characteristic resistance phenotype in barley plants (*mlo*‐38 and *mlo*‐29, respectively; Lundqvist *et al*., 1991; Piffanelli *et al*., 2002; Müller *et al*., 2005; Reinstädler *et al*., 2010). Furthermore, a similar mutation to *Tamlo‐A1* (P325L) was studied by transient gene expression in barley (P324A), showing a partial resistance phenotype (Reinstädler *et al*., 2010). Analysis with the online tool SIFT (Sorting Intolerant From Tolerant; Kumar *et al*., 2009) revealed in each genome at least one mutation predicted to affect protein function (Table 2). Taken together, we identified 16 missense mutations, distributed between all three homoeologues that would potentially affect *Ta*Mlo function. Since nonsense mutations were not identified (Table 1), we further functionally characterized the missense mutations (Table 2) to select the most suitable candidates for stacking of the alleles in single wheat lines.

**Table 1:** Mutations in the region of exon 9 of *TaMlo* identified by TILLING

Target	Total candidates	False positives	Intron	Silent	Missense	Non sense
*TaMlo‐A1*	28	9	7	10	2	0
*TaMlo‐B1*	27	4	5	11	7	0
*TaMlo‐D1*	21	7	2	5	7	0

**Table 2:** *TaMlo* exon 9 missense mutants identified by TILLING

Gene	EMS line	SNP		Amino acid exchange	Zygosity^a^	SIFT score^b^	Previously described in barley
		WT	Mutant				
*TaMlo‐A1*	CAD1‐29‐A8	C	T	P325L	Het	0.20	*mlo* P324A Reinstädler *et al*. (2010)
*TaMlo‐A1*	CAD‐22‐D12	C	T	A354V	Hom	0.00*	No
*TaMlo‐B1*	CAD1‐2‐H9	G	A	G296E	Het	0.00*	No
*TaMlo‐B1*	CAD2‐18‐H1	C	T	T297I	Het	0.04	No
*TaMlo‐B1*	CAD2‐22‐E11	C	T	R313W	Het	0.01*	No
*TaMlo‐B1*	CAD2‐1‐A3	G	A	S315N	Het	0.49	No
*TaMlo‐B1*	CAD2‐21‐D7	G	A	G319R	Hom	0.00*	*mlo*‐38 Lundqvist *et al*. (1991), Reinstädler *et al*. (2010)
*TaMlo‐B1*	CAD1‐4‐H10	G	A	A320T	Het	0.97	No
*TaMlo‐B1*	CAD1‐2‐G9	C	T	T345M	Het	0.09	No
*TaMlo‐D1*	CAD2‐1‐A2	G	A	A314T	Het	0.65	No
*TaMlo‐D1*	CAD2‐19‐C9	G	A	G319R	Het	0.01*	*mlo*‐38 Lundqvist *et al*. (1991), Reinstädler *et al*. (2010)
*TaMlo‐D1*	CAD2‐23‐A2	G	A	A320T	Het	1.00	No
*TaMlo‐D1*	CAD2‐24‐C8	C	T	P321S	Het	0.25	No
*TaMlo‐D1*	CAD2‐19‐H4	G	A	V323I	Hom	0.13	No
*TaMlo‐D1*	CAD2‐3‐B6	C	T	P335L	Hom	0.00*	*mlo*‐29 Piffanelli *et al*. (2002), Müller *et al*. (2005)
*TaMlo‐D1*	CAD2‐29‐A1	C	T	T345M	Hom	0.09	No

a Hom, Homozygous; Het, Heterozygous.

b SIFT scores ≤0.05 indicate mutations that are predicted to affect protein functions (indicated by an asterisk), while SIFT scores >0.05 indicate mutations that are predicted to be tolerated.

### Validation of mutant candidates by transient gene expression

Previously, we demonstrated that *TaMlo‐B1* is able to complement a powdery mildew‐resistant barley *mlo* mutant at the single‐cell level in leaf epidermal tissue (Elliott *et al*., 2002). Heterologous complementation is possible because of the high level of sequence identity between the wheat and barley Mlo proteins (~89%). We took advantage of this particle bombardment‐based barley transient expression assay to experimentally evaluate the potential of the *Ta*Mlo variants with amino acid substitutions to alter the powdery mildew infection phenotype.

Full‐length cDNAs of *TaMlo‐A1*,* TaMlo‐B1* and *TaMlo‐D1* were cloned as expression constructs driven by the maize *Ubiquitin1* promoter (pUbiGATE) and used as a template to recreate each of the 16 missense mutations by PCR‐based, site‐directed mutagenesis. In addition, WT barley *Mlo* cDNA (also driven by the *Ubiquitin1* promoter) was used to generate the mutant variants P324A, *mlo*‐29 (P334L) and *mlo*‐38 (G318R), which were included as additional controls.

**Figure 2:**
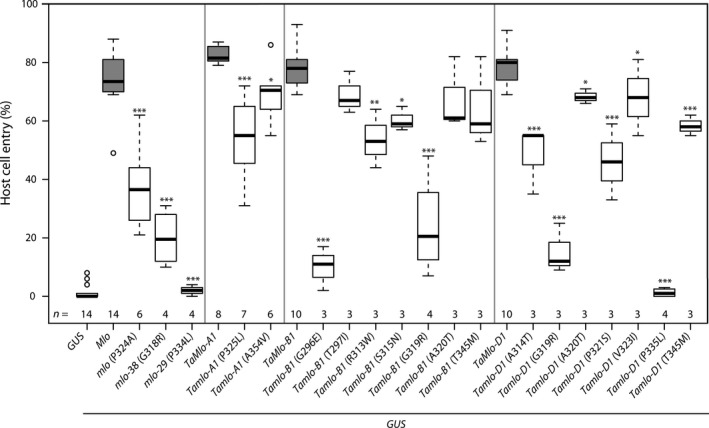
TILLING‐derived variants of *Tamlo* homoeologues exhibit different levels of functionality in a transient gene expression assay. Detached 8‐day‐old leaves of the powdery mildew‐resistant barley *mlo*‐3 mutant were co‐bombarded with a *GUS* reporter plasmid and a plasmid encoding the indicated *Ta*Mlo protein variant (WT or mutant version under transcriptional control of the maize *Ubiquitin1* promoter). Expression of *GUS* alone and *GUS* plus WT barley *Mlo*, driven by the maize *Ubiquitin1* promoter, were used as negative and positive controls, respectively, for restoration of *Bgh* susceptibility. Host cell entry was scored at 48 hours post inoculation (h p.i.) in GUS‐stained cells attacked by powdery mildew sporelings and results visualized as box plots. Centre lines show the medians; upper and lower box limits indicate the 25th and 75th percentiles respectively; upper and lower whiskers extend 1.5 times the interquartile range from the 25th and 75th percentiles, respectively, and outliers are represented by dots. Numbers at the bottom of the boxplots indicate the number of biological replicates per sample (*n*). One biological replicate was typically composed of six leaves with 150 scored cells. Asterisks indicate a statistically significant difference to the respective WT (barley Mlo or *Ta*Mlo) with ***P <* *0.001, **P <* *0.01 and *P <* *0.5 as determined by a Generalized Linear Model (GLM) test. Statistics were performed and boxplots generated with R software.

Detached leaves of the barley *mlo*‐3 null mutant were co‐bombarded with the various *TaMlo* or *Mlo* constructs and a plasmid expressing the *β‐glucuronidase* (*GUS*) reporter gene. As a negative control we employed the *GUS* plasmid only, and as a positive control we used barley *Mlo*. Twenty‐four hours after bombardment the treated leaves were inoculated with the barley powdery mildew pathogen *Bgh*. All three WT wheat *TaMlo* genes showed a complementation efficiency similar to barley *Mlo* (~80% host cell entry, median value, Figure 2). Transient expression of mutants *Tamlo‐A1* (P325L), its barley counterpart *mlo* (P324A), *Tamlo‐B1* (R313W) and *Tamlo‐D1* (A314T), (P321S) and (T345M) resulted in ~40%–60% host cell entry (median value, Figure 2). Expression of mutants *Tamlo‐B1* (G296E) and *Tamlo‐B1*,* Tamlo‐D1* (G319R) led to an even lower host cell entry rate of ~15%–20% (Figure 2). Similar results were obtained with the corresponding G318R barley mutant (*mlo*‐38, Figure 2). Interestingly, the entry rates obtained by expression of *Tamlo‐D1* (P335L) and the corresponding barley mutant variant (*mlo*‐29) were close to background levels (*GUS* only, Figure 2), suggesting a near‐complete loss‐of‐function in the case of these amino acid substitutions. Expression of the remaining seven wheat mutant variants showed host cell entry rates not considerably different from the respective *TaMlo* WT versions (Figure 2). Overall there was a good correlation between the results of the *in silico* SIFT analysis and the experimental data. Exceptions were mutations *Tamlo‐A1* (A354V), *Tamlo‐B1* (T297I) and *Tamlo‐B1* (R313W), which were predicted to have a significant impact on protein function but were only moderately affected regarding host cell entry rates (Figure 2 and Table 2).

### Barley mutants *mlo*‐29 and *mlo*‐38 as a case study to compare results from transient gene expression assays and resistance *in planta*

**Figure 3:**
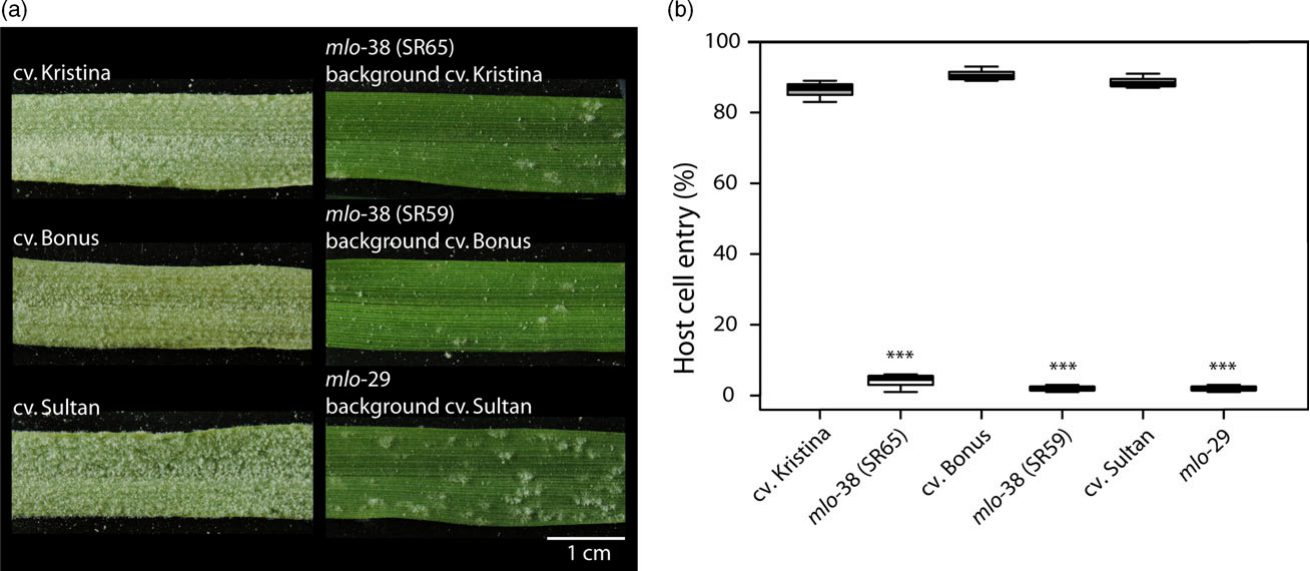
*Bgh* infection phenotypes of barley mutants *mlo*‐38 and *mlo*‐29 and their respective parental lines. Seven‐day‐old leaves were inoculated with *Bgh* isolate K1. (a) The macroscopic phenotype was recorded at 7 days post inoculation (d.p.i.). A scale bar shown in white is given in the lower right corner (1 cm). (b) Host cell entry was scored at 48 h p.i. Centre lines show the medians; upper and lower box limits indicate the 25th and 75th percentiles respectively; upper and lower whiskers extend 1.5 times the interquartile range from the 25th and 75th percentiles, respectively. Shown are data of *n* = 3 independent biological replicates with 3–4 leaves (typically 200 scored interaction sites per leaf) per replicate. Asterisks indicate a statistically significant difference to the respective parental background with ***P <* *0.001 as determined by a GLM test. Statistics were performed and boxplots established by R software.

Since we found wheat equivalents of barley mutants *mlo*‐29 (P335L) and *mlo*‐38 (G319R) in our wheat TILLING screening, we compared the powdery mildew host cell entry rate *in planta* (which has not been assessed before; Figure 3) with the one obtained in transient expression experiments (Figure 2). The respective WT barley parental lines cv. Bonus, Kristina and Sultan showed high susceptibility to *Bgh* (Figure 3a) with ~85% host cell entry (Figure 3b). By contrast, the mutants *mlo*‐38 (two lines: SR59, background cv. Bonus and SR65, background cv. Kristina) and *mlo*‐29 (background cv. Sultan) were highly resistant to the pathogen (Figure 3a) with a host cell entry rate of less than 6% (Figure 3b). At least in the case of *mlo*‐38, this value is substantially lower than the one obtained upon transient expression of the *mlo*‐38 variant (~20% entry rate, median value; Figure 2). These results suggest that overexpression of *mlo* mutant variants may result in an overestimation of the residual protein function (*i.e*. artificially high protein levels may partly compensate for the defect in the protein). Consequently, for a given *mlo* mutant allele the resulting level of resistance *in planta* can be higher (*i.e*. respective plants are more resistant) than indicated by the results of the single‐cell transient gene expression assays.

### Generation of triple homozygous *Tamlo* lines

Based on the data obtained with transient gene expression experiments (Figure 2), mutant candidates that showed reduced host cell entry compared to the respective *TaMlo* WT genes were selected for allele stacking (Table 3). While several suitable mutants with moderately or strongly reduced *Ta*Mlo function were available for the wheat genomes B and D, only two mutants were available for the A genome, which conferred moderately (P325L) and slightly (A354V) reduced host cell entry in the transient gene expression assay (Figure 2 and Table 3). Due to the lack of alternatives for genome A, mutant P325L was used in all crossings. With the aim of balancing the degree of powdery mildew resistance and potential pleiotropic phenotypes associated with the loss of *Mlo* function (Hückelhoven *et al*., 2013), we generated four wheat lines containing different mutant allele combinations that ranged from predicted weak to strong effects on powdery mildew susceptibility (Table 3). Depending on the zygosity of the available single mutants identified in the TILLING screen we followed a crossing scheme similar to the one shown in Figure S1 to obtain ideally all possible mutant combinations. To identify the respective mutations in the progeny of the crossings we developed cleaved amplified polymorphic sequence (CAPS) markers (Table S2). Genotype confirmation was subsequently performed by DNA sequencing of the 2nd round PCR products of the particular *TaMlo* homoeologues.

**Table 3:** Triple mutant wheat lines obtained by stacking of *Tamlo* mutant alleles

Line No.	*Tamlo‐A1*		*Tamlo‐B1*		*Tamlo‐D1*		Current status
	Mutation	Effect^a^	Mutation	Effect^a^	Mutation	Effect^a^	
1	P325L	Medium	G319R	Strong	P335L	Very strong	*aabbdd*
2	P325L	Medium	G319R	Strong	G319R	Strong	*aabbdd*
3	P325L	Medium	G296E	Strong	P321S	Medium	*AaBbDd*
4	P325L	Medium	T297I	Weak	P321S	Medium	*AaBbDd*

a Effect on powdery mildew susceptibility based on data from transient gene expression assays (Figure 2).

Currently, we have produced four different *Tamlo* lines that bear mutations in all three *TaMlo* homoeologues. Lines 1 and 2 (Table 3) are already homozygous and are referred as *Tamlo‐aabbdd*. We also obtained two of the three possible double mutant combinations (*Tamlo‐aabbDD* and *Tamlo‐AAbbdd*). Lines 3 and 4 (Table 3) are presently triple heterozygous plants in self‐fertilization stage.

### Mutations in *TaMlo* homoeologues confer enhanced *Bgt* resistance

**Figure 4:**
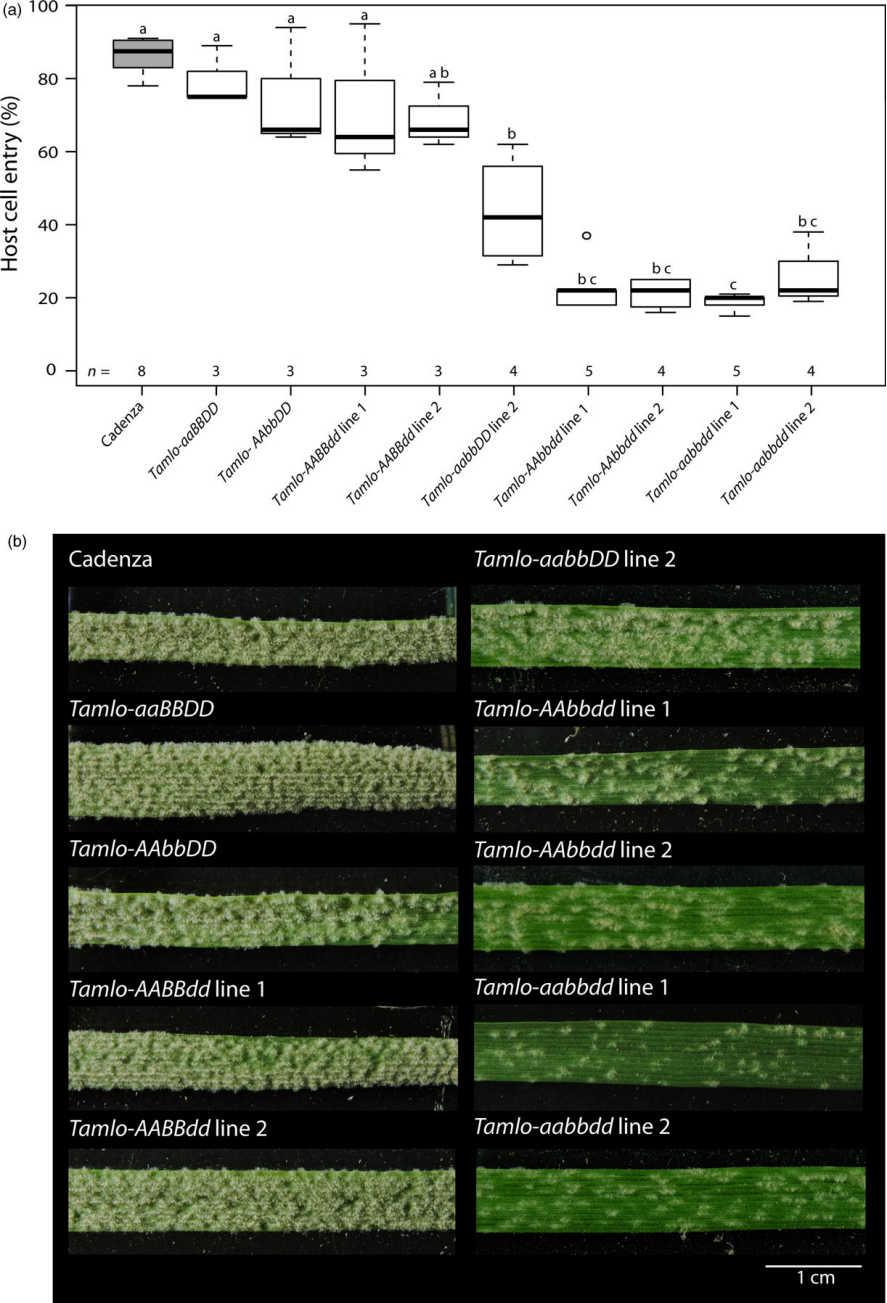
*Bgt* infection phenotypes of wheat WT (cv. Cadenza), single, double and triple *Tamlo* mutants. Ten‐day‐old leaves were inoculated with *Bgt* isolate JA82. (a) Host cell entry was scored at 72 h p.i. Centre lines show the medians; upper and lower box limits indicate the 25th and 75th percentiles respectively; upper and lower whiskers extend 1.5 times the interquartile range from the 25th and 75th percentiles, respectively, and outliers are represented by dots. Numbers at the bottom of the boxplots indicate the number of biological replicates per sample (*n*). One biological replicate was typically composed of four leaves with 200 scored cells. Letters indicate genotypes whose data are significantly (*P *<* *0.05) different from genotypes labelled with other letters, as determined by pair‐wise testing with a Games–Howell *post hoc* test. Statistics were performed and boxplots generated with R software. (b) First leaves of germinated seedlings were fixed with surgical tape to a polycarbonate platform and inoculated with *Bgt* conidiospores. The macroscopic phenotype was recorded at 6 d.p.i. A scale bar shown in white is given in the lower right corner (1 cm).

We next aimed to test the powdery mildew infection phenotype with the set of available single, double and triple mutants. We inoculated 10‐day‐old leaves with a *Bgt* field isolate and estimated microscopically the host cell entry rate at 72 h p.i. (hours post inoculation; Figure 4a). Furthermore, we evaluated the macroscopic phenotype of these mutants at 6 h p.i. (hours post inoculation; Figure 4b).

The cv. Cadenza exhibited a *Bgt* penetration rate of ~85%. The various single homozygous mutants showed a slight reduction in entry rate (~65%–75%, not significantly different from cv. Cadenza), with the tendency of *Tamlo‐AAbbDD* and *Tamlo‐AABBdd* to be lower than *Tamlo‐aaBBDD;* Figure 4a). Interestingly, both *Tamlo‐AAbbdd* double mutant lines showed a lower penetration rate than the *Tamlo‐aabbDD* double mutant line (between 14% and 22% for *Tamlo‐AAbbdd versus* 42% for *Tamlo‐aabbDD*, Figure 4a and S2a; note that *Tamlo‐aabbDD* was obtained from segregating progeny in line 2, but is based on the same allele combination as in line 1). Furthermore, both *Tamlo*‐*aabbdd* triple mutant lines exhibited enhanced resistance compared with WT, with host cell entry values of ~20%, similar to the *Tamlo*‐*AAbbdd* double mutant (Figures 4a and S2a). This result suggests that the contribution of the *Tamlo‐A1* allele to resistance in the triple homozygous line is lower than the contribution of the *Tamlo‐B1* and ‐*D1* alleles, as was predicted by the transient expression data (Figure 2).

The evaluation of the macroscopic phenotype upon *Bgt* inoculation was largely consistent with the microscopic assessment (Figures 4b and S2b). The four single mutants exhibited an infection phenotype similar to WT, while the *Tamlo‐aabbDD* and *Tamlo‐AAbbdd* double mutant lines and the two *Tamlo‐aabbdd* triple mutant lines showed enhanced resistance compared to the WT (Figures 4b and S2b). However, in contrast to the host cell entry results, at the level of the macroscopic infection phenotype the triple *Tamlo‐aabbdd* mutants seem to be more resistant than the *Tamlo‐AAbbdd* and *Tamlo‐aabbDD* double mutants, as exemplified by fewer colonies on the leaf surface (Figures 4b and S2b). This observation suggests that the *Tamlo‐aabbdd* lines might have an additional post‐penetration effect in powdery mildew infection. Nevertheless, resistance of each mutant was clearly weaker than seen for the triple knockout mutant obtained by TALENs (Wang *et al*., 2014; Figures 4b and S2b).

Although resistance of the TILLING mutants in the field has not yet been scored, plants were naturally and spontaneously infected by *Bgt* in the greenhouse. Throughout the entire wheat growth cycle we observed considerably fewer powdery mildew symptoms on the *Tamlo‐aabbdd* lines compared to the WT or heterozygous individuals within segregating populations (Figure S3).

### Differential transcript accumulation of the *TaMlo1* homoeologues

**Figure 5:**
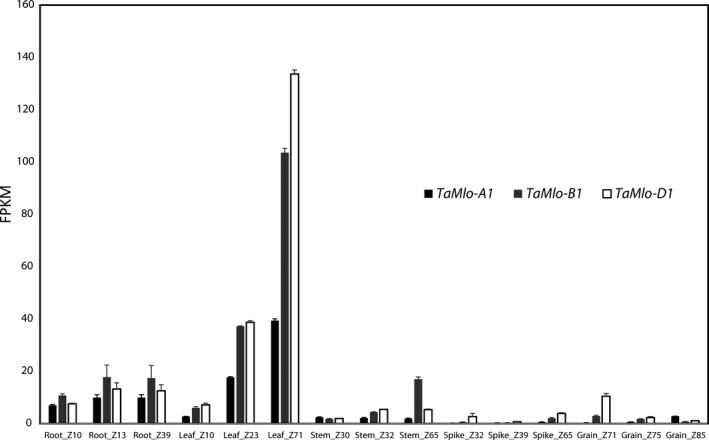
Transcript accumulation of *TaMlo* homoeologues in five bread wheat tissues at different developmental stages. Transcript levels of each homoeologue were summed up and then averaged across replicates, ± standard errors of the mean. FPKM: Fragments per kb per million mapped reads; Z: Zadoks developmental scale (Zadoks *et al*., 1974).

Since we observed a higher reduction in the *Bgt* penetration rate and less sporulation on the leaf surface of the *Tamlo‐AAbbdd* double mutant compared to the *Tamlo‐aabbDD* double mutant (Figure 4) we reasoned that this difference could be based (i) on the particular mutant allele combinations; (ii) on potential unequal functional redundancy of the *TaMlo* homoeologues; or (iii) on differential expression of the three *TaMlo* homoeologues. To explore the latter possibility, we made use of a data set of RNA‐seq samples derived from several tissues (root, leaf, stem, spike and grain) at different developmental stages of bread wheat cv. Chinese Spring (Choulet *et al*., 2014). The paired‐end RNA‐seq reads were mapped to a transcriptome reference containing the coding sequences of *TaMlo* obtained in this study (File S1) together with non‐redundant cDNA sequences from The International Wheat Genome Sequencing Consortium (IWGSC, 2014). The respective mean fragments per kilobase (kb) per million mapped reads (FPKM) values revealed that the *TaMlo* homoeologues are highly expressed in leaves compared to the other tissues, especially at Zadoks stage 71 (Zadoks *et al*., 1974; Figure 5 and Table S3). Furthermore, the FPKM values suggest that in leaves the three homoeologues have differential expression levels, with *TaMlo‐D1* showing the highest level of expression, followed by *TaMlo‐B1*, while the level of *TaMlo‐A1* transcripts seems strikingly lower (Figure 5).

To further test the notion that *TaMlo‐A1* shows lower transcript levels in leaf tissue compared to *TaMlo‐B1* and ‐*D1*, we followed an entirely independent experimental route. We PCR‐amplified and cloned a *TaMlo* cDNA fragment using oligonucleotide primers that have a complete match in all three *TaMlo* homoeologues. This cDNA fragment was further chosen to contain several homoeologue‐specific single‐nucleotide polymorphisms (SNPs) that distinguish them unequivocally (Figure S4). We randomly picked and sequenced 28 clones representing the various *TaMlo* cDNA fragments. Among these, we found 14, 10 and 4 clones corresponding to *TaMlo‐B1, TaMlo‐D1* and *TaMlo‐A1*, respectively. Together the results of these two approaches suggest that *TaMlo‐A1* is the homoeologue with the lowest transcript levels in leaves.

### TILLING‐derived *Tamlo* triple mutant lines lack obvious pleiotropic phenotypes

In our conditions, the *Tamlo‐aabbdd* lines did not show any obvious and macroscopically discernible differences regarding overall growth habit, height, the number of spikes or seed number compared to cv. Cadenza WT plants. We also did not notice any signs of early leaf chlorosis in the TILLING mutants compared to the WT. This is in stark contrast to the pronounced leaf chlorosis observed under our growth conditions in case of the TALEN‐derived *Tamlo‐aabbdd* line, which represents a full *Tamlo* knockout mutant (Wang *et al*., 2014, Figure S5).

## Discussion

In this study, we used the non‐transgenic TILLING technology as a strategy to identify mutant candidates in the *TaMlo* genes from an EMS‐mutagenized population of spring wheat cv. Cadenza. TILLING has been previously suggested as a means to create mutants in ‘susceptibility genes’ such as *Mlo* in a targeted manner (Hückelhoven *et al*., 2013; van Schie and Takken, 2014). By using transient gene expression assays in barley and an adapted powdery mildew species for the bioassay we were able to test the obtained missense mutant wheat variants and to select candidates that confer different degrees of reduction in powdery mildew host cell entry (Figure 2). This experimental assessment of *Ta*Mlo protein functionality broadly agreed with the results of *in silico* prediction *via* SIFT analysis (Figure 2 and Table 2). However, the observed exceptions [*Tamlo‐A1* (A354V), *Tamlo‐B1* (T297I) and *Tamlo‐B1* (R313W)] highlight the need for validation of mutant variants by experimentation. Stacking of the selected mutant alleles resulted in four different wheat lines carrying a missense mutation in each of the three *TaMlo* homoeologues (Table 3). The two triple homozygous *Tamlo‐aabbdd* mutant lines (1 and 2) showed enhanced powdery mildew disease resistance*,* exemplified by a considerable reduction in *Bgt* host cell entry [from ~85% in WT to ~20% (median values) in the triple mutant] and a substantial decrease in macroscopically visible *Bgt* colonies (Figures 4 and S2). Notably, the *Tamlo‐AAbbdd* double mutants showed a similar level of enhanced *Bgt* penetration resistance.

In our TILLING screening we targeted *TaMlo* exon 9, which encodes the third cytoplasmic loop of the *Ta*Mlo protein. Excluding the false positives, we found a total of 56 mutants within an approximate population of 2020 individuals (Table 1), which represents an average density of 1 mutation per 32 kb screened. These results are comparable to the ones obtained by Botticella and co‐workers after screening the same TILLING population for mutants in the *SbIIa* genes (Botticella *et al*., 2011). These authors identified an average of 60 candidate mutants per exon and a density of approximately 1 mutation per 40 kb screened. As in our screening, the authors did not find nonsense mutations for each exon assessed. However, in contrast to our results, the number of identified missense mutations was higher than the silent ones. The lack of identification of nonsense mutations in our study is somewhat surprising because exon 9 has six glutamine (Q; CAG triplet) or tryptophan (W; TGG triplet) codons that could potentially have been mutated to stop codons.

Using transient gene expression experiments in single barley epidermal cells we demonstrated that cDNAs from each of the three *TaMlo* homoeologues are able to complement the barley *mlo* resistance phenotype and can restore host cell entry to WT‐like levels (Figure 2). Of the 16 identified missense mutations, the non‐conservative changes had a more dramatic effect on susceptibility to powdery mildew, especially G296E, G319R and P335L (Figure 2 and Table 3). These data are consistent with previous site‐directed mutagenesis studies performed in barley, further reinforcing the relevance of the third cytoplasmic loop of Mlo as an important domain in the susceptibility‐conferring activity of the protein (Reinstädler *et al*., 2010). Therefore, the application of transient gene expression assays was an efficient tool to select suitable mutant alleles for subsequent stacking.

Surprisingly, not only the *Tamlo‐aabbdd* triple mutants but also the double mutants *Tamlo‐aabbDD* and *Tamlo‐AAbbdd* exhibited significantly enhanced *Bgt* resistance compared to the WT (Figures 4 and S2). These results are in stark contrast with the ones obtained by Wang and co‐workers, where only the triple knockout mutant exhibited a notable difference in the powdery mildew infection phenotype (Wang *et al*., 2014). However, the *Tamlo‐AAbbdd* double mutant line was missing in the set of tested TALEN plants (Wang *et al*., 2014); therefore, a comparison between this double mutant and ours generated by TILLING is not possible. It is conceivable that some of the discrepancies between the two data sets are due to the type of evaluation, since we scored host cell entry while Wang and co‐workers evaluated the number of microcolonies per germinated spores (Wang *et al*., 2014). Additionally, second‐site mutations present in our TILLING lines, which are derived from EMS mutagenesis, might lead to differences in the powdery mildew infection phenotype. Furthermore, the TILLING lines are based on a spring wheat cultivar (cv. Cadenza), while the TALEN lines are derived from a winter wheat. Together, our results challenge the hypothesis of strict functional redundancy between wheat *TaMlo* homoeologues proposed by Borrill *et al*. (2015) and suggest instead that the powdery mildew infection phenotype can be the result of gene dosage effects or differential expression of the respective *TaMlo* homoeologues. Evidence to support this notion is provided not only by the differences in host cell entry and macroscopic infection phenotypes observed between the double mutants *Tamlo‐aabbDD* and *Tamlo‐AAbbdd* compared to WT (Figure 4), but also the seemingly lower transcript levels of *TaMlo‐A1* compared to *TaMlo‐B1* and ‐*D1* (Figure 5). *Tamlo* double mutants with enhanced powdery mildew resistance might be advantageous in future breeding programmes, since only two mutant alleles must be followed in segregating populations.

The incomplete *Bgt* resistance found in our *Tamlo‐aabbdd* triple mutants is likely based on the partial loss‐of‐function alleles used in these lines (Figure 2; Table 3). Deployment of yet to be identified stronger EMS‐induced alleles (e.g. nonsense mutations) may, similar to the TALEN‐derived line (Wang *et al*., 2014), result in a TILLING‐based non‐transgenic triple mutant with full *Bgt* resistance. However, the incomplete resistance phenotype in our generated *Tamlo‐aabbdd* TILLING lines might in fact represent an advantage in several different ways, namely, reduced pleiotropic effects, more durable resistance and an opportunity for breeders since products of induced mutagenesis are free of government regulation.

Pleiotropic effects such as early leaf senescence associated with *mlo* mutants have been better studied in barley where lines carrying weak resistance alleles, permitting residual fungal growth, exhibit fewer pleiotropic phenotypes than *mlo* mutants harbouring strong alleles, which confer complete immunity (Hentrich, 1979; Piffanelli *et al*., 2002). TILLING has thus been suggested as a means to find partial loss‐of‐function mutants of ‘susceptibility genes’ that show mild pleiotropy (Hückelhoven *et al*., 2013). During the first cycle of propagation we did not observe differences in growth, development and fertility between our *Tamlo‐aabbdd* TILLING lines (partial loss‐of‐function alleles) and the WT, nor did we observe enhanced or premature leaf senescence (Figure S5). These results are in contrast to the noticeable leaf chlorosis in TALEN‐based mutants (complete loss‐of‐function alleles) in comparison to its parental line KN199 (Figure S5). Nevertheless, this is only a first qualitative assessment of these plants and quantitative measurements of photosynthetic performance and chlorophyll levels under various growth conditions are needed to substantiate these observations in future experiments.

The most common allele introduced in the spring barley varieties cultivated in Europe is *mlo‐*11 (Brown and Rant, 2013; Kokina *et al*., 2007; Piffanelli *et al*., 2004). Barley plants carrying this natural partial loss‐of‐function allele are not fully resistant and allow the growth of powdery mildew colonies at a low level (Piffanelli *et al*., 2004). The barley *mlo*‐11 plants nevertheless confer broad‐spectrum resistance to the pathogen, which has been durable in the field for more than 30 years (reviewed in Acevedo‐Garcia *et al*., 2014). One may speculate that the lower selection pressure exerted on powdery mildew populations by partial resistance might have contributed to this durability under agricultural conditions. Our *Tamlo‐aabbdd* lines are also partially resistant, allowing a low level of *Bgt* sporulation (Figure 4). This feature may contribute to the robustness of the trait once *mlo*‐based resistance is used in commercial wheat crops. Finally, our non‐transgenic wheat *Tamlo‐aabbdd* plants represent an excellent alternative for breeders and farmers in regions where growth of genetically‐modified organisms (GMOs) is banned and where regulations for the cultivation of genome‐edited crops are still under discussion (Chen *et al*., 2014b; Huang *et al*., 2016).

This study reports the first step towards the generation of new commercial bread wheat varieties with an enhanced resistance phenotype to the globally important powdery mildew disease, generated by TILLING technology targeting the *TaMlo* gene. Currently, our *Tamlo‐aabbdd* plants are being backcrossed to remove excess of EMS mutations that may affect additional traits. It will be interesting to determine the performance of the backcrossed plants in the field at the agronomic level and their response to other pathogens and pests that attack wheat crops.

## Experimental procedures

### Plant and fungal material

The EMS‐mutagenized population of the spring wheat (*Triticum aestivum*) cv. Cadenza has been previously described (Rakszegi *et al*., 2010). Barley (*Hordeum vulgare*) seeds NGB14661.2 (cv. Kristina), NGB14682.1 (cv. Bonus) NGB119178.1 (SR59, *mlo*‐38, background cv. Bonus), NGB119185.1 (SR65, *mlo*‐38, background cv. Kristina) were originally reported in Lundqvist *et al*. (1991) and obtained from NordGen (Nordic Genetic Resource Centre) in Sweden. The *mlo*‐29 mutant and its parent cv. Sultan were described previously (Piffanelli *et al*., 2002). The TALEN‐derived *Tamlo‐aabbdd* line and its parental line KN199 (Wang *et al*., 2014) were kindly provided by Prof. Caixia Gao and Prof. Jin‐Long Qiu from the State Key Laboratories (Chinese Academy of Sciences, Beijing, China). The *Bgt* isolate, termed JA82, was obtained from plant material spontaneously infected in the greenhouse, latitude: 50.7784, longitude: 6.04863. It was propagated weekly on 10‐day‐old seedlings comprising a mixture of summer wheat cv. Munk and commercially available seeds of unknown identity. *Bgh* isolate K1 was propagated weekly on 7‐day‐old barley seedlings cv. Margret.

### TILLING screening

M_2_ wheat genomic DNA samples (~2020) of the TILLING library developed at Rothamsted Research were twofold pooled in 96‐well plates at 25 ng/μL. The amplicons for HRM were produced by a nested PCR strategy. The 1st round PCR was carried out in 10 μL final volume using 1 μL of pooled template DNA, 5 μL of HotShot Diamond^™^ Master Mix (Clent Life Sciences, Stourbridge, UK) and 0.5 μm primers. The PCR program was: 98 °C, 5 min; (97 °C, 30 s; 66–67 °C, 30 s; 72 °C 1 min) × 35 cycles; 72 °C, 5 min; 10 °C until end.

For HRM, the 1st round PCR reaction was diluted 100‐fold and 2 μL were used as template for the 2nd round PCR. The 2nd round PCR was prepared in FrameStar 96‐well skirted plates, black frame with white wells (4titude, Surrey, UK). The reaction contained 2 μL of diluted DNA template, 0.5 μm of each primer, 5 μL HotShot Diamond Mastermix, and 1 μL of LCGreen Plus (Clent Life Sciences, Stourbridge, UK) in a total volume of 10 μL; 10 μL of mineral oil (M5904, Sigma, Deisenhofen, Germany) was added to prevent evaporation. The PCR program was similar to the primary PCR, with annealing at 66 °C and after final extension, an additional denaturation step at 98 °C, 3 min, followed by cooling to 10 °C at 0.2 °C/s.

### High‐Resolution Melting analysis

The 2nd round PCR plates were used for HRM analysis using the LightScanner instrument (Idaho Technology Inc., Utah) and with the following temperatures: start, 82 °C; end 98 °C and a hold of 78 °C. The analysis was performed with the software provided with the scanner and as described in Botticella *et al*. (2011). Negative samples were repeated and positive candidates were selected from the independent plate. A twofold pool of DNA from candidate lines together with cv. Cadenza DNA was generated for the 2nd round PCR and screening was repeated. The amplicon from putative positive candidates was re‐amplified and sent for sequencing. DNA sequencing was performed by Eurofins MWG Operon (Ebersberg, Germany).

### Transient gene expression in barley leaf epidermal cells

Ballistic transformation of 8‐day‐old detached barley *mlo*‐3 (background cv. Ingrid) leaves was performed as previously described (Reinstädler *et al*., 2010). Inoculation with *Bgh* isolate K1 was carried out at 24 h after bombardment and histochemical staining with X‐Gluc (5‐Bromo‐4‐chloro‐3‐indolyl‐ß‐D‐glucoronide; Carl Roth GmbH, Karlsruhe, Germany) for β‐glucoronidase (GUS) activity was performed at 48 h p.i. Fungal structures were stained with Coomassie Brilliant Blue R‐250 (Carl Roth GmbH, Karlsruhe, Germany). The host cell entry rate was calculated as: (number of transformed, *Bgh*‐attacked cells with haustoria/transformed and *Bgh*‐attacked cells)*100. At least six leaves were evaluated per mutant with a minimum of 150–200 cells per experiment and at least three independent biological replicates.

For further experimental details, see Files S3 and S4.

## Supporting information


**Figure S1** Crossing scheme to obtain the *Tamlo‐aabbdd* line 1. The procedure started with the propagation of M_2_ seeds and the selection of single homozygous mutants for each *TaMlo* homoeologue (*Tamlo‐aaBBDD*,* Tamlo‐AAbbDD* and *Tamlo‐AABBdd*). The homozygous single mutant lines *Tamlo‐AAbbDD* and *Tamlo‐AABBdd* were crossed, the resulting heterozygous F_1_ progeny (genotype *Tamlo‐AABbDd*) self‐fertilized, and homozygous *Tamlo‐AAbbdd* double mutant plants selected in the F_2_ generation. These were finally crossed with the homozygous *Tamlo‐aaBBDD* single mutant, the resulting heterozygous F_1_ progeny (genotype *Tamlo‐AaBbDd*) self‐fertilized, and homozygous *Tamlo‐aabbdd* triple mutant plants selected in the F_2_ generation. A crossing is represented with an *x* and self‐pollination is represented by an *x* inside a circle. Similar crossing schemes were adapted for the other allele combinations.

**Figure S2** *Bgt* infection phenotypes of additional independent *Tamlo* mutant lines and the TALEN‐derived *Tamlo‐aabbdd* mutant with its respective parent. Ten‐day‐old leaves were inoculated with *Bgt* conidiospores. (a) Host cell entry was scored at 72 h p.i. Centre lines show the medians; upper and lower box limits indicate the 25th and 75th percentiles, respectively; upper and lower whiskers extend 1.5 times the interquartile range from the 25th and 75th percentiles respectively. Numbers at the bottom of the boxplots indicate the number of biological replicates per sample (*n*). One biological replicate was typically composed of four leaves with 200 scored cells. Letters indicate genotypes whose data are significantly (*P *<* *0.001) different from genotypes labelled with other letters, as determined by pair‐wise testing with a Games–Howell *post hoc* test. Statistics were performed and boxplots generated with R software. (b) First leaves of germinated seedlings were fixed with surgical tape to a polycarbonate platform and inoculated with *Bgt* conidiospores. The macroscopic phenotype was recorded at 6 d.p.i. A scale bar shown in white is given in the lower right corner (1 cm).

**Figure S3** Spontaneous powdery mildew infection of wheat plants growing under greenhouse conditions. (a) cv. Cadenza. (b) Heterozygous plant of segregating population, genotype *Tamlo‐AaBBdd*. (c–d) *Tamlo‐aabbdd* line 1. (a and c) 2‐month‐old, (b and d) 3‐month‐old plants.

**Figure S4** Nucleotide sequences of the *TaMlo* cDNA fragment chosen for indirect *TaMlo* expression analysis by PCR product cloning. The sequence represents the PCR fragment obtained with oligonucleotide primers TaMlo‐ABD‐F and TaMlo‐ABD‐R (Table S1), indicated with bold lines. Shown in yellow are SNPs for *TaMlo‐A1*, in blue SNPs for *TaMlo‐B1*, in green SNPs for *TaMlo‐D1* and in grey a SNP for all three homoeologues.

**Figure S5** Early leaf senescence phenotype of the TALEN‐derived *Tamlo‐aabbdd* line**.** (a) Transgenic winter wheat TALEN line (genotype *Tamlo‐aabbdd*; left) and its parental line WT KN199 (genotype *Tamlo‐AABBDD*; right). (b) TILLING‐derived spring wheat line 1 (genotype *Tamlo‐aabbdd*; left) and its WT parent cv. Cadenza (genotype *Tamlo‐AABBDD*; right). (c) TILLING‐derived spring wheat line 2 (genotype *Tamlo‐aabbdd*; left) and its WT parent cv. Cadenza (genotype *Tamlo‐AABBDD*; right). All plants were around 3 months old when images were taken.

**Table S1** Sequences of oligonucleotide primers used in this study.

**Table S2** CAPS markers developed for genotyping *Tamlo* mutants.

**Table S3** Homoeologue‐specific *TaMlo* gene transcript levels in wheat tissues.

**File S1** Multiple sequence alignment of *TaMlo* coding sequences.

**File S2** *TaMlo* consensus genomic sequences.

**File S3** Supplementary Materials and Methods.

**File S4** R script for the Games–Howell *post hoc* test.
