# Supplementary figures and images for: mlo‐based powdery mildew resistance in hexaploid bread wheat generated by a non‐transgenic TILLING approach
Source: Plant Biotechnol J. 2016 Sep 25;15(3):367–78. doi: 10.1111/pbi.12631 (PMC5316926; doi:10.1111/pbi.12631)

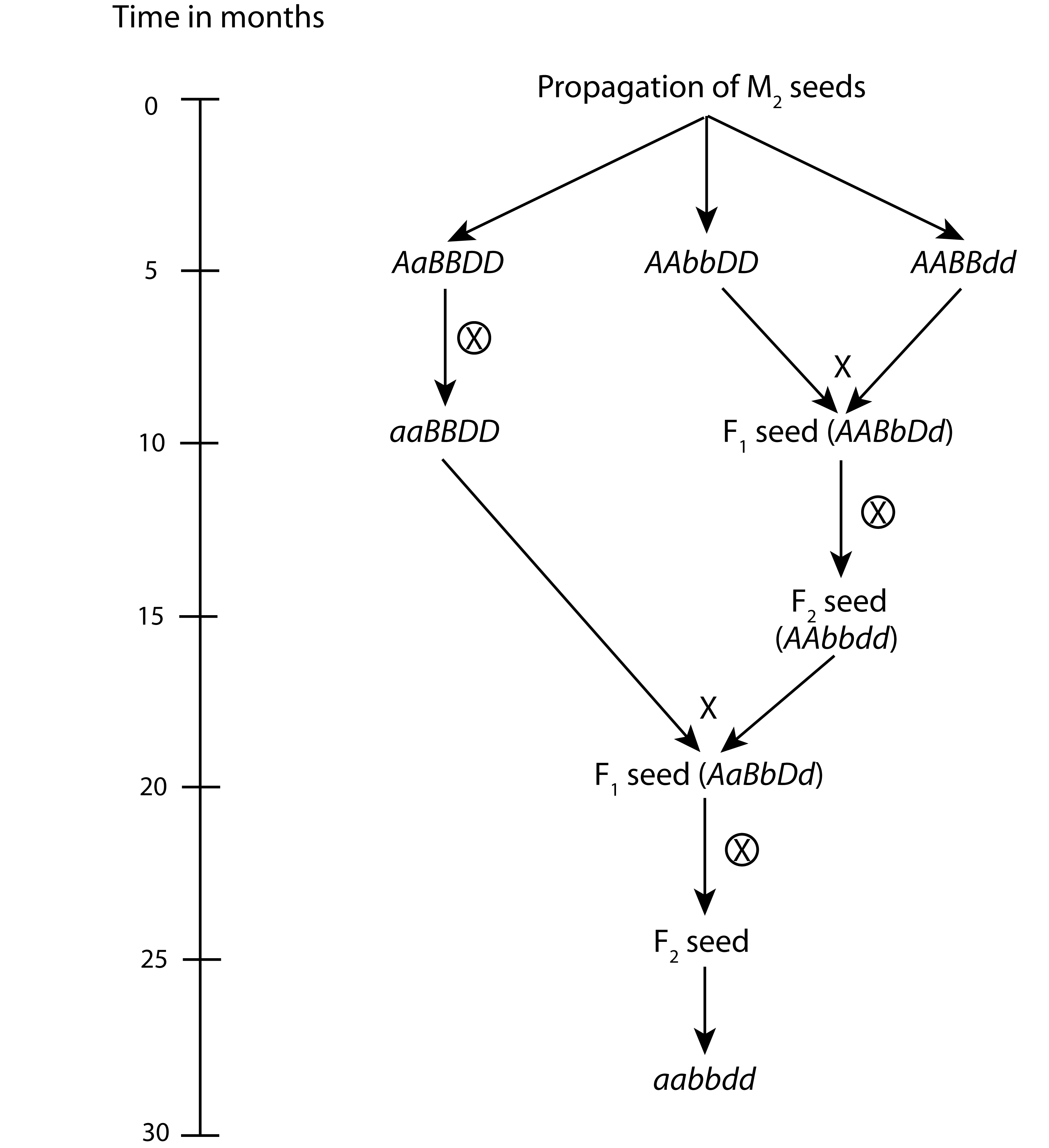

Supplement: Supplementary file 1 — Figure S1 Crossing scheme to obtain the Tamlo‐aabbdd line 1. The procedure started with the propagation of M2 seeds and the selection of single homozygous mutants for each TaMlo homoeologue (Tamlo‐aaBBDD, Tamlo‐AAbbDD and Tamlo‐AABBdd). The homozygous single mutant lines Tamlo‐AAbbDD and Tamlo‐AABBdd were crossed, the resulting heterozygous F1 progeny (genotype Tamlo‐AABbDd) self‐fertilized, and homozygous Tamlo‐AAbbdd double mutant plants selected in the F2 generation. These were finally crossed with the homozygous Tamlo‐aaBBDD single mutant, the resulting heterozygous F1 progeny (genotype Tamlo‐AaBbDd) self‐fertilized, and homozygous Tamlo‐aabbdd triple mutant plants selected in the F2 generation. A crossing is represented with an x and self‐pollination is represented by an x inside a circle. Similar crossing schemes were adapted for the other allele combinations. [file PBI-15-367-s009.png]

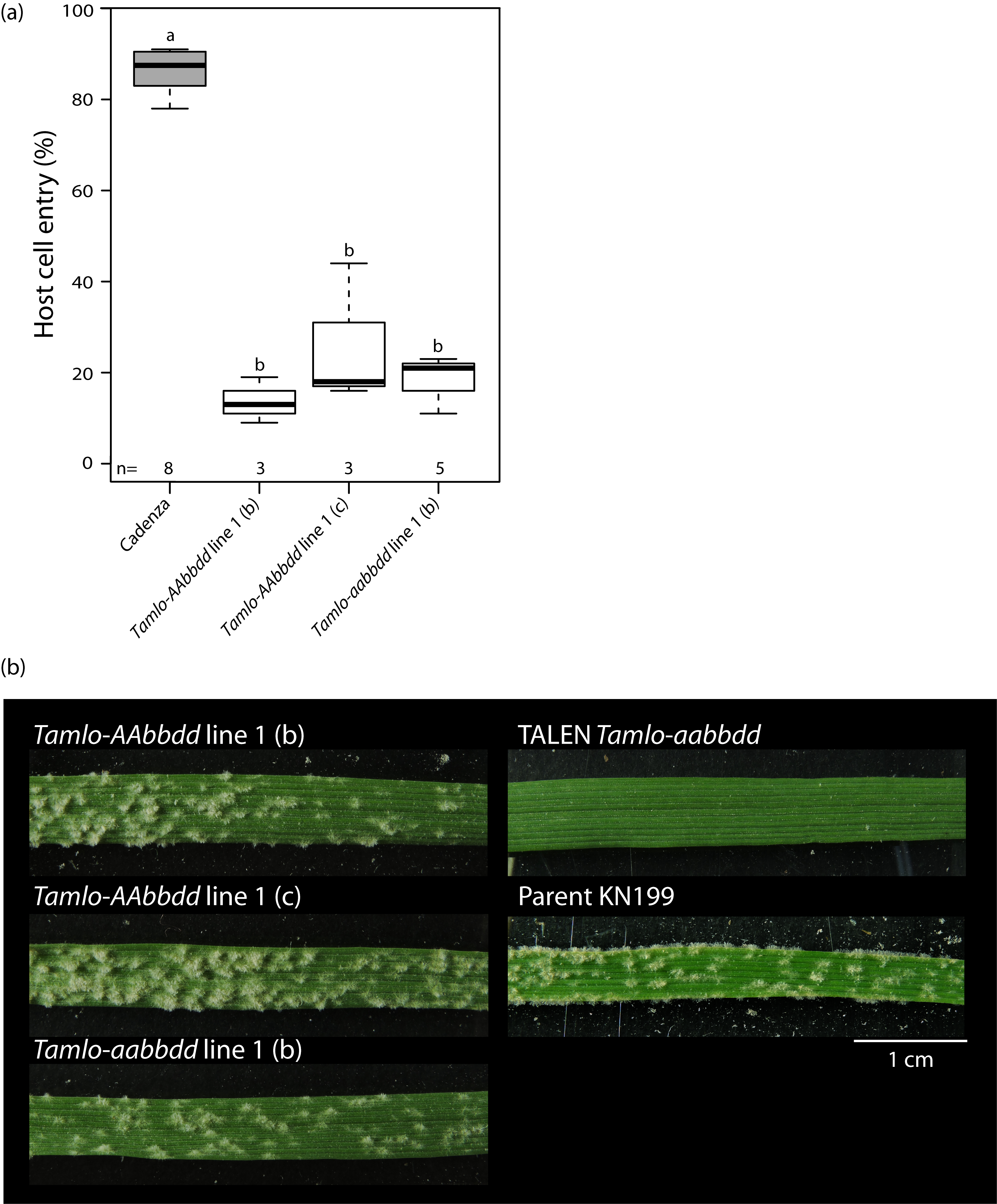

Supplement: Supplementary file 2 — Figure S2 Bgt infection phenotypes of additional independent Tamlo mutant lines and the TALEN‐derived Tamlo‐aabbdd mutant with its respective parent. Ten‐day‐old leaves were inoculated with Bgt conidiospores. (a) Host cell entry was scored at 72 h p.i. Centre lines show the medians; upper and lower box limits indicate the 25th and 75th percentiles, respectively; upper and lower whiskers extend 1.5 times the interquartile range from the 25th and 75th percentiles respectively. Numbers at the bottom of the boxplots indicate the number of biological replicates per sample (n). One biological replicate was typically composed of four leaves with 200 scored cells. Letters indicate genotypes whose data are significantly (P < 0.001) different from genotypes labelled with other letters, as determined by pair‐wise testing with a Games–Howell post hoc test. Statistics were performed and boxplots generated with R software. (b) First leaves of germinated seedlings were fixed with surgical tape to a polycarbonate platform and inoculated with Bgt conidiospores. The macroscopic phenotype was recorded at 6 d.p.i. A scale bar shown in white is given in the lower right corner (1 cm). [file PBI-15-367-s008.png]

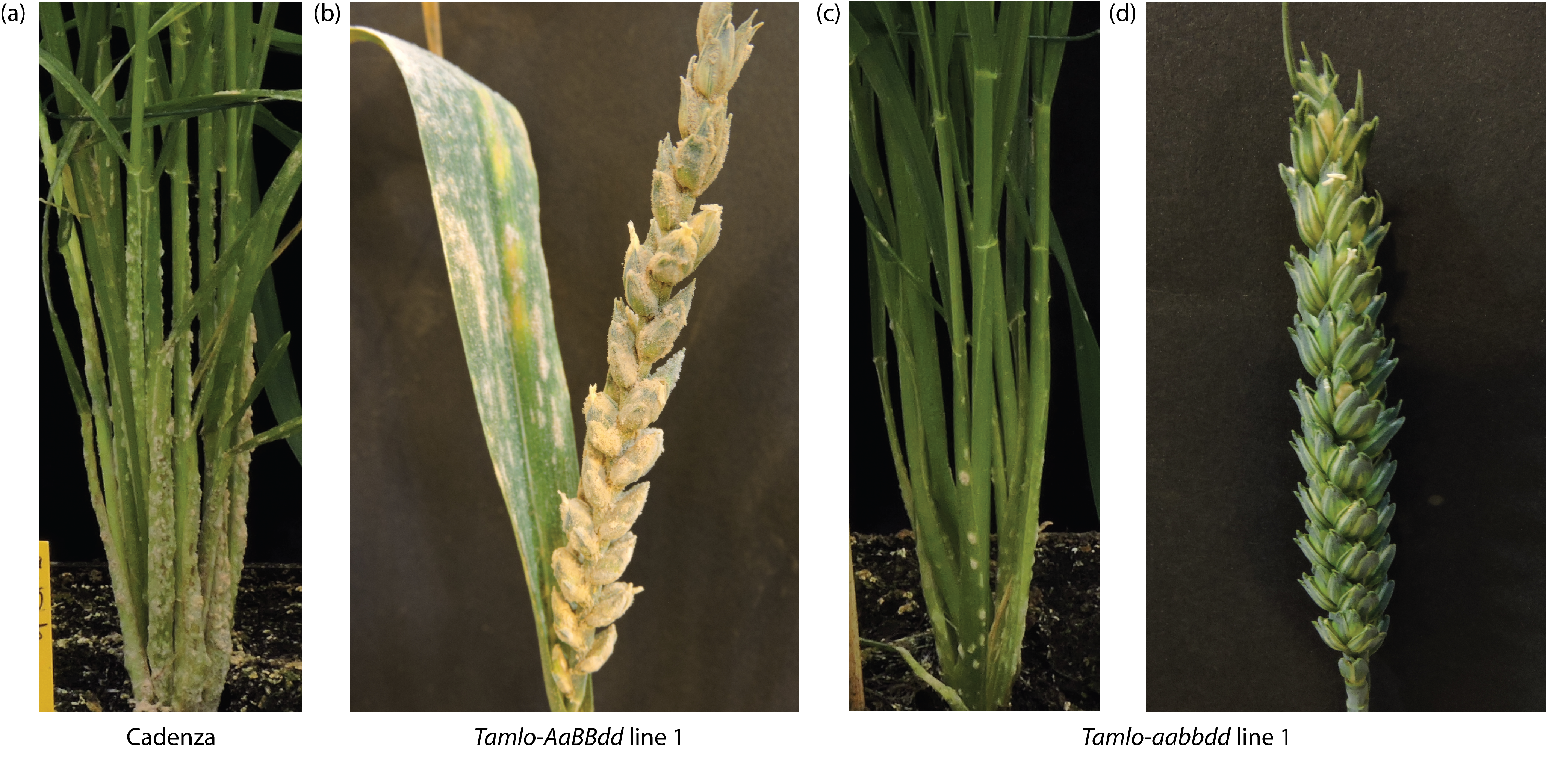

Supplement: Supplementary file 3 — Figure S3 Spontaneous powdery mildew infection of wheat plants growing under greenhouse conditions. (a) cv. Cadenza. (b) Heterozygous plant of segregating population, genotype Tamlo‐AaBBdd. (c–d) Tamlo‐aabbdd line 1. (a and c) 2‐month‐old, (b and d) 3‐month‐old plants. [file PBI-15-367-s007.png]

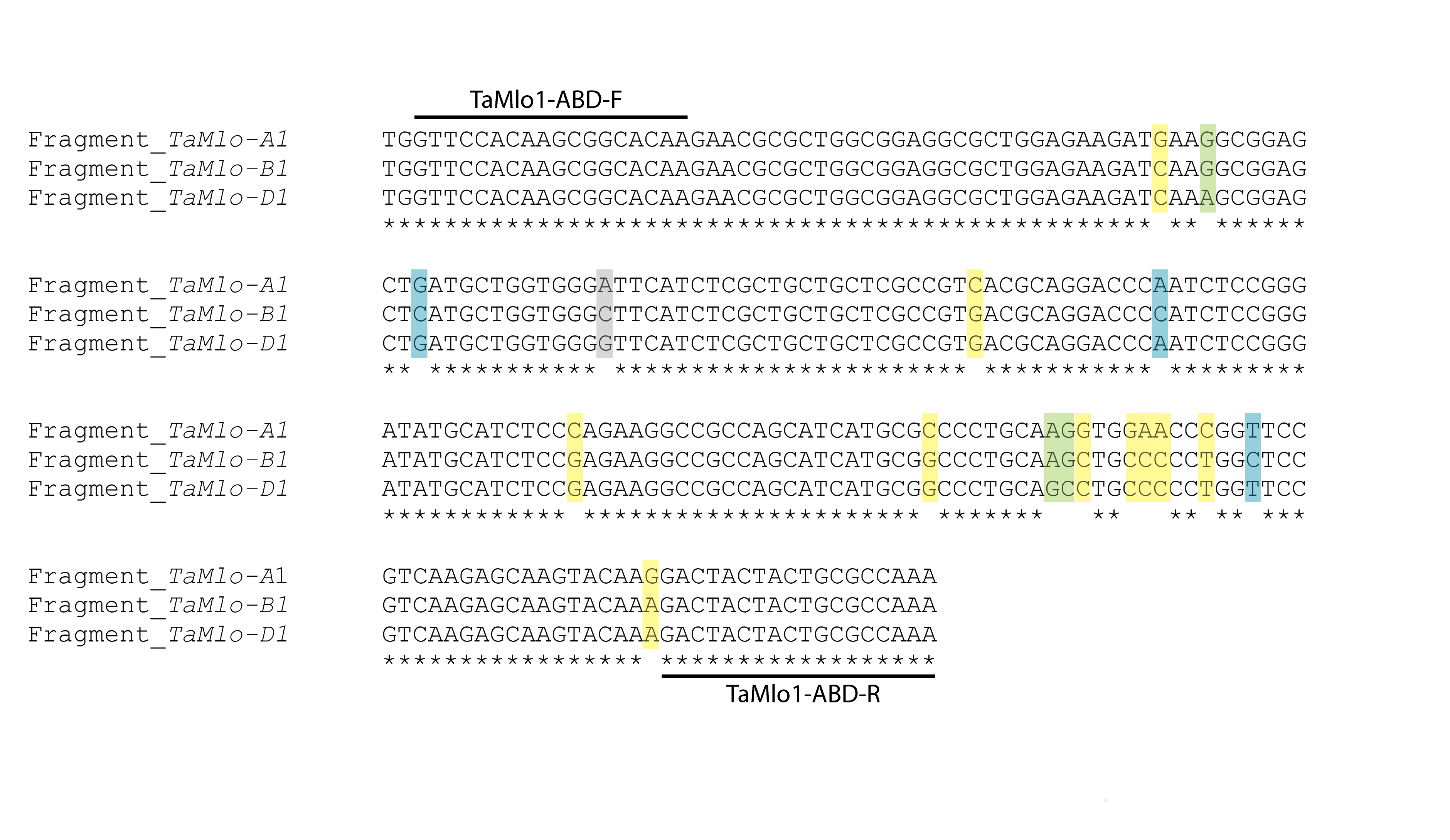

Supplement: Supplementary file 4 — Figure S4 Nucleotide sequences of the TaMlo cDNA fragment chosen for indirect TaMlo expression analysis by PCR product cloning. The sequence represents the PCR fragment obtained with oligonucleotide primers TaMlo‐ABD‐F and TaMlo‐ABD‐R (Table S1), indicated with bold lines. Shown in yellow are SNPs for TaMlo‐A1, in blue SNPs for TaMlo‐B1, in green SNPs for TaMlo‐D1 and in grey a SNP for all three homoeologues. [file PBI-15-367-s011.png]

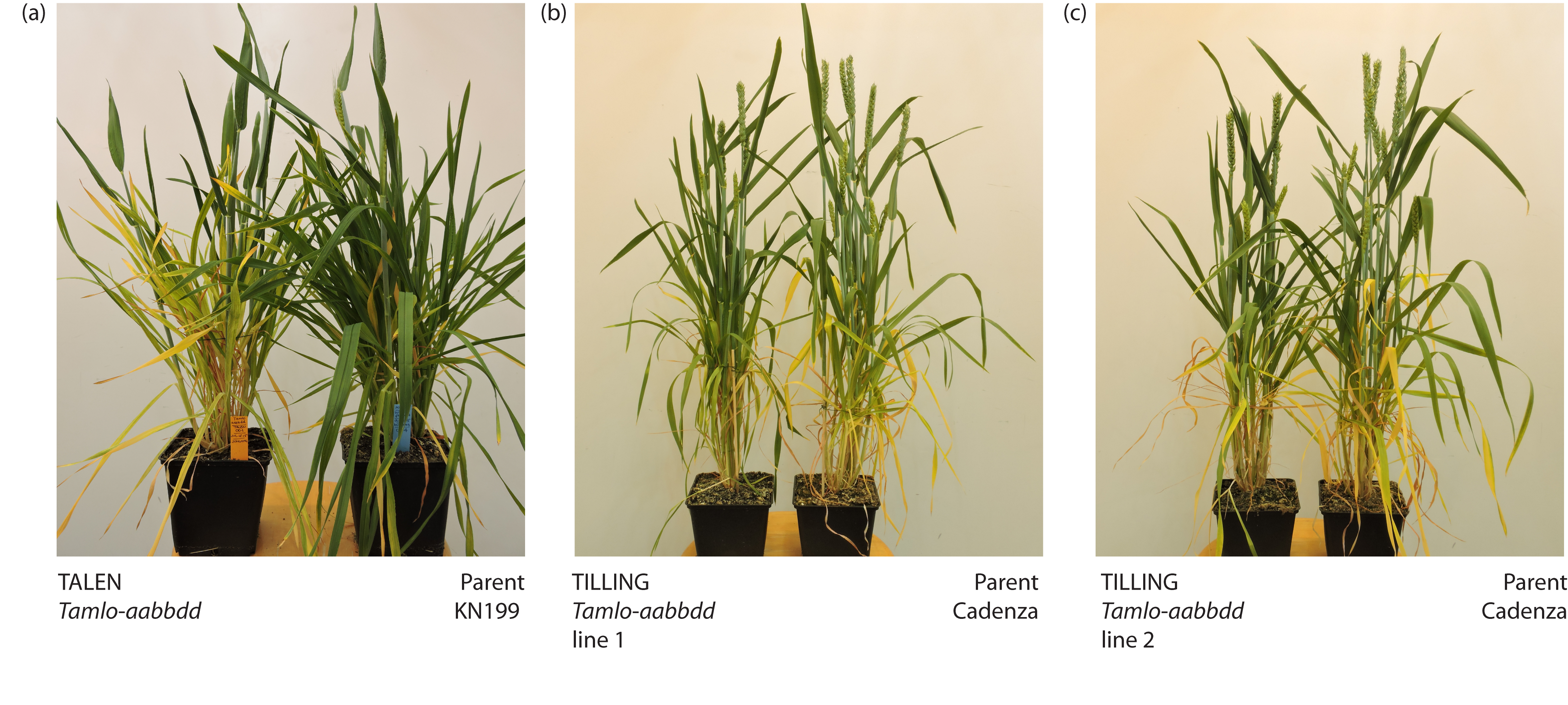

Supplement: Supplementary file 5 — Figure S5 Early leaf senescence phenotype of the TALEN‐derived Tamlo‐aabbdd line. (a) Transgenic winter wheat TALEN line (genotype Tamlo‐aabbdd; left) and its parental line WT KN199 (genotype Tamlo‐AABBDD; right). (b) TILLING‐derived spring wheat line 1 (genotype Tamlo‐aabbdd; left) and its WT parent cv. Cadenza (genotype Tamlo‐AABBDD; right). (c) TILLING‐derived spring wheat line 2 (genotype Tamlo‐aabbdd; left) and its WT parent cv. Cadenza (genotype Tamlo‐AABBDD; right). All plants were around 3 months old when images were taken. [file PBI-15-367-s001.png]
