# Supplementary material for: mlo‐based powdery mildew resistance in hexaploid bread wheat generated by a non‐transgenic TILLING approach
Source: Plant Biotechnol J. 2016 Sep 25;15(3):367–78. doi: 10.1111/pbi.12631 (PMC5316926; doi:10.1111/pbi.12631)
Supplement: Supplementary file 9 — File S1 Multiple sequence alignment of TaMlo coding sequences. [file PBI-15-367-s005.docx]

**File S1** Multiple sequence alignment of *TaMlo* coding sequences.

TaMlo-A1 ATGGCAAAGGACGACGGGTACCCCCCGGCGCGGACGCTGCCGGAGACGCCGTCCTGGGCG

TaMlo-B1 ATGGCGGACGACGACGAGTACCCCCCAGCGAGGACGCTGCCGGAGACGCCGTCCTGGGCG

TaMlo-D1 ATGGCGGAGGACTACGAGTACCCCCCGGCGCGGACGCTGCCGGAGACGCCGTCCTGGGCG

***** * *** *** ********* *** *****************************

TaMlo-A1 GTGGCGCTGGTCTTCGCCGTCATGATCATCGTCTCCGTCCTCCTGGAGCACGCGCTCCAC

TaMlo-B1 GTGGCCCTCGTCTTCGCCGTCATGATCATCGTGTCCGTCCTCCTGGAGCACGCGCTCCAT

TaMlo-D1 GTGGCGCTCGTCTTCGCCGTCATGATCATCGTGTCCGTCCTCCTGGAGCACGCGCTCCAC

***** ** *********************** **************************

TaMlo-A1 AAGCTCGGCCATTGGTTCCACAAGCGGCACAAGAACGCGCTGGCGGAGGCGCTGGAGAAG

TaMlo-B1 AAGCTCGGCCATTGGTTCCACAAGCGGCACAAGAACGCGCTGGCGGAGGCGCTGGAGAAG

TaMlo-D1 AAGCTCGGCCATTGGTTCCACAAGCGGCACAAGAACGCGCTGGCGGAGGCGCTGGAGAAG

************************************************************

TaMlo-A1 ATGAAGGCGGAGCTGATGCTGGTGGGATTCATCTCGCTGCTGCTCGCCGTCACGCAGGAC

TaMlo-B1 ATCAAGGCGGAGCTCATGCTGGTGGGCTTCATCTCGCTGCTGCTCGCCGTGACGCAGGAC

TaMlo-D1 ATCAAAGCGGAGCTGATGCTGGTGGGGTTCATCTCGCTGCTGCTCGCCGTGACGCAGGAC

** ** ******** *********** *********************** *********

TaMlo-A1 CCAATCTCCGGGATATGCATCTCCCAGAAGGCCGCCAGCATCATGCGCCCCTGCAAGGTG

TaMlo-B1 CCCATCTCCGGGATATGCATCTCCGAGAAGGCCGCCAGCATCATGCGGCCCTGCAAGCTG

TaMlo-D1 CCAATCTCCGGGATATGCATCTCCGAGAAGGCCGCCAGCATCATGCGGCCCTGCAGCCTG

** ********************* ********************** ******* **

TaMlo-A1 GAACCCGGTTCCGTCAAGAGCAAGTACAAGGACTACTACTGCGCCAAAGAGGGCAAGGTG

TaMlo-B1 CCCCCTGGCTCCGTCAAGAGCAAGTACAAAGACTACTACTGCGCCAAACAGGGCAAGGTG

TaMlo-D1 CCCCCTGGTTCCGTCAAGAGCAAGTACAAAGACTACTACTGCGCCAAAAAGGGCAAGGTG

** ** ******************** ****************** ***********

TaMlo-A1 GCGCTCATGTCCACGGGCAGCCTGCACCAGCTCCACATATTCATCTTCGTGCTAGCCGTC

TaMlo-B1 TCGCTCATGTCCACGGGCAGCTTGCACCAGCTGCACATATTCATCTTCGTGCTCGCCGTC

TaMlo-D1 TCGCTAATGTCCACGGGCAGCTTGCACCAGCTCCACATATTCATCTTCGTGCTCGCCGTC

**** *************** ********** ******************** ******

TaMlo-A1 TTCCATGTCACCTACAGCGTCATCATCATGGCTCTAAGCCGTCTCAAGATGAGAACATGG

TaMlo-B1 TTCCATGTCACCTACAGCGTCATCATCATGGCTCTAAGCCGTCTCAAAATGAGAACCTGG

TaMlo-D1 TTCCATGTCACCTACAGCGTCATCATCATGGCTCTAAGCCGTCTCAAAATGAGGACATGG

*********************************************** ***** ** ***

TaMlo-A1 AAGAAATGGGAGACAGAGACCGCCTCCTTGGAATACCAGTTCGCAAATGATCCTGCGCGG

TaMlo-B1 AAGAAATGGGAGACAGAGACCGCCTCCCTGGAATACCAGTTCGCAAATGATCCTGCGCGG

TaMlo-D1 AAGAAATGGGAGACAGAGACCGCCTCCTTGGAATACCAGTTCGCAAATGATCCTGCGCGG

*************************** ********************************

TaMlo-A1 TTCCGCTTCACGCACCAGACGTCGTTCGTGAAGCGGCACCTGGGCCTGTCCAGCACCCCC

TaMlo-B1 TTCCGCTTCACGCACCAGACGTCGTTCGTGAAGCGGCACCTGGGCCTCTCCAGCACCCCC

TaMlo-D1 TTCCGCTTCACGCACCAGACGTCGTTCGTGAAGCGTCACCTGGGCCTCTCCAGCACCCCC

*********************************** *********** ************

TaMlo-A1 GGCGTCAGATGGGTGGTGGCCTTCTTCAGGCAGTTCTTCAGGTCGGTCACCAAGGTGGAC

TaMlo-B1 GGCGTCAGATGGGTGGTGGCCTTCTTCAGGCAGTTCTTCAGGTCGGTCACCAAGGTGGAC

TaMlo-D1 GGCATCAGATGGGTGGTGGCCTTCTTCAGGCAGTTCTTCAGGTCGGTCACCAAGGTGGAC

*** ********************************************************

TaMlo-A1 TACCTCACCTTGAGGGCAGGCTTCATCAACGCGCACTTGTCGCAGAACAGCAAGTTCGAC

TaMlo-B1 TACCTCACCTTGAGGGCAGGCTTCATCAACGCGCATTTGTCGCATAACAGCAAGTTCGAC

TaMlo-D1 TACCTCACCCTGAGGGCAGGCTTCATCAACGCGCATTTGTCGCATAACAGCAAGTTCGAC

********* ************************* ******** ***************

TaMlo-A1 TTCCACAAGTACATCAAGAGGTCCATGGAGGACGACTTCAAAGTCGTCGTTGGCATCAGC

TaMlo-B1 TTCCACAAGTACATCAAGAGGTCCATGGAGGACGACTTCAAAGTCGTCGTTGGCATCAGC

TaMlo-D1 TTCCACAAGTACATCAAGAGGTCCATGGAGGACGACTTCAAAGTCGTCGTTGGCATCAGC

************************************************************

TaMlo-A1 CTCCCGCTGTGGGCTGTGGCGATCCTCACCCTCTTCCTTGATATCGACGGGATCGGCACA

TaMlo-B1 CTCCCGCTGTGGTGTGTGGCGATCCTCACCCTCTTCCTTGACATTGACGGGATCGGCACG

TaMlo-D1 CTCCCGCTGTGGTGTGTGGCGATCCTCACCCTCTTCCTTGATATTGACGGGATCGGCACG

************ *************************** ** **************

TaMlo-A1 CTCACCTGGGTTTCTTTCATCCCTCTCATCATCCTCTTGTGTGTTGGAACCAAGCTAGAG

TaMlo-B1 CTCACCTGGATTTCTTTCATCCCTCTCGTCATCCTCTTGTGTGTTGGAACCAAGCTGGAG

TaMlo-D1 CTCACCTGGATTTCTTTCATCCCTCTCGTCATCCTCTTGTGTGTTGGAACCAAGCTGGAG

********* ***************** **************************** ***

TaMlo-A1 ATGATCATCATGGAGATGGCCCTGGAGATCCAGGACCGGTCGAGCGTCATCAAGGGGGCA

TaMlo-B1 ATGATCATCATGGAGATGGCCCTGGAGATCCAGGACCGGGCGAGCGTCATCAAGGGGGCG

TaMlo-D1 ATGATCATCATGGAGATGGCCCTGGAGATCCAGGACCGGGCGAGCGTCATCAAGGGGGCG

*************************************** *******************

TaMlo-A1 CCCGTGGTCGAGCCCAGCAACAAGTTCTTCTGGTTCCACCGCCCCGACTGGGTCCTCTTC

TaMlo-B1 CCCGTGGTTGAGCCCAGCAACAAGTTCTTCTGGTTCCACCGCCCCGACTGGGTCCTCTTC

TaMlo-D1 CCCGTGGTTGAGCCCAGCAACAAGTTCTTCTGGTTCCACCGCCCCGACTGGGTCCTCTTC

******** ***************************************************

TaMlo-A1 TTCATACACCTGACGCTGTTCCAGAACGCGTTTCAGATGGCACATTTCGTGTGGACAGTG

TaMlo-B1 TTCATACACCTGACGCTATTCCAGAACGCGTTTCAGATGGCACATTTCGTGTGGACAGTG

TaMlo-D1 TTCATACACCTGACGCTGTTCCAGAATGCGTTTCAGATGGCACATTTCGTCTGGACAGTG

***************** ******** *********************** *********

TaMlo-A1 GCCACGCCCGGCTTGAAGGACTGCTTCCATATGAACATCGGGCTGAGCATCATGAAGGTC

TaMlo-B1 GCCACGCCCGGCTTGAAGAAATGCTTCCATATGCACATCGGGCTGAGCATCATGAAGGTC

TaMlo-D1 GCCACGCCCGGCTTGAAGAAATGCTTCCATATGCACATCGGTCTGAGCATCATGAAGGTC

****************** * ************ ******* ******************

TaMlo-A1 GTGCTGGGGCTGGCTCTCCAGTTCCTGTGCAGCTACATCACCTTCCCCCTCTACGCGCTA

TaMlo-B1 GTGCTGGGGCTGGCTCTTCAGTTCCTCTGCAGCTATATCACCTTCCCGCTCTACGCGCTC

TaMlo-D1 GTGCTGGGGCTGGCTCTTCAGTTCCTCTGCAGCTATATCACCTTCCCCCTCTACGCGCTC

***************** ******** ******** *********** ***********

TaMlo-A1 GTCACACAGATGGGATCAAACATGAAGAGGTCCATCTTCGACGAGCAGACAGCCAAGGCG

TaMlo-B1 GTCACACAGATGGGATCAAACATGAAGAGGTCCATCTTCGACGAGCAGACGGCCAAGGCG

TaMlo-D1 GTCACACAGATGGGATCGAACATGAAGAGGTCCATCTTCGACGAGCAGACGGCCAAGGCG

***************** ******************************** *********

TaMlo-A1 CTGACCAACTGGCGGAACACGGCCAAGGAGAAGAAGAAGGTCCGAGACACGGACATGCTG

TaMlo-B1 CTGACAAACTGGCGGAACACGGCCAAGGAGAAGAAGAAGGTCCGAGACACGGACATGCTG

TaMlo-D1 CTGACCAACTGGCGGAACACGGCCAAGGAGAAGAAGAAGGTCCGAGACACGGACATGCTG

***** ******************************************************

TaMlo-A1 ATGGCGCAGATGATCGGCGACGCAACACCCAGCCGAGGCACGTCCCCGATGCCTAGCCGG

TaMlo-B1 ATGGCGCAGATGATCGGCGACGCGACGCCCAGCCGAGGGGCGTCGCCCATGCCTAGCCGG

TaMlo-D1 ATGGCGCAGATGATCGGCGACGCGACGCCCAGCCGAGGCACGTCGCCGATGCCTAGCCGG

*********************** ** *********** **** ** ************

TaMlo-A1 GGCTCATCGCCGGTGCACCTGCTTCAGAAGGGCATGGGACGGTCTGACGATCCCCAGAGC

TaMlo-B1 GGCTCGTCGCCAGTGCACCTGCTTCACAAGGGCATGGGACGGTCCGACGATCCCCAGAGC

TaMlo-D1 GCTTCGTCACCGGTGCACCTGCTTCACAAGGGCATGGGACGGTCCGACGATCCCCAGAGC

* ** ** ** ************** ***************** ***************

TaMlo-A1 GCACCGACCTCGCCAAGGACCATGGAGGAGGCTAGGGACATGTACCCGGTTGTGGTGGCG

TaMlo-B1 ACGCCAACCTCGCCAAGGGCCATGGAGGAGGCTAGGGACATGTACCCGGTTGTGGTGGCG

TaMlo-D1 GCGCCGACCTCGCCAAGGACCATGGAGGAGGCTAGGGACATGTACCCGGTTGTGGTGGCG

* ** ************ *****************************************

TaMlo-A1 CATCCTGTACACAGACTAAATCCTGCTGACAGGCGGAGGTCGGTCTCTTCATCAGCCCTC

TaMlo-B1 CATCCAGTGCACAGACTAAATCCTGCTGACAGGAGAAGGTCGGTCTCGTCGTCGGCACTC

TaMlo-D1 CATCCCGTGCACAGACTAAATCCTGCTGACAGGCGGAGGTCGGTCTCTTCGTCGGCACTC

***** ** ************************ * *********** ** ** ** ***

TaMlo-A1 GATGCCGACATCCCCAGCGCAGATTTTTCCTTCAGCCAGGGATGA

TaMlo-B1 GATGTCGACATTCCCAGCGCAGATTTTTCCTTCAGCCAGGGATGA

TaMlo-D1 GATGCCGACATCCCCAGCGCAGATTTTTCCTTCAGCCAGGGATGA

**** ****** *********************************
