# Supplementary material for: mlo‐based powdery mildew resistance in hexaploid bread wheat generated by a non‐transgenic TILLING approach
Source: Plant Biotechnol J. 2016 Sep 25;15(3):367–78. doi: 10.1111/pbi.12631 (PMC5316926; doi:10.1111/pbi.12631)
Supplement: Supplementary file 10 — File S2 TaMlo consensus genomic sequences. [file PBI-15-367-s012.docx]

**File S2** *TaMlo* consensus genomic sequences.

Exons are shown in capital letters and intron in small letters

>TaMlo-A1

ATGGCAAAGGACGACGGGTACCCCCCGGCGCGGACGCTGCCGGAGACGCC

GTCCTGGGCGGTGGCGCTGGTCTTCGCCGTCATGATCATCGTCTCCGTCC

TCCTGGAGCACGCGCTCCACAAGCTCGGCCATgtaagtcccctcactccc

gcaacaagaacaagaacaagaacaaccagaaccagaatcagctcatggct

tcctttcctcccttggtgcgtgtaagcagTGGTTCCACAAGCGGCACAAG

AACGCGCTGGCGGAGGCGCTGGAGAAGATGAAGGCGGAGCTGATGCTGGT

GGGATTCATCTCGCTGCTGCTCGCCGTCACGCAGGACCCAATCTCCGGGA

TATGCATCTCCCAGAAGGCCGCCAGCATCATGCGCCCCTGCAAGGTGGAA

CCCGGTTCCGTCAAGAGCAAGTACAAGGACTACTACTGCGCCAAAgaggt

aactaacacaaacagtttcttcttcttcttcttgttgttttccttcctga

ttggcttggcctgattggtgtggtgtctgtttctcctgGAGGGCAAGGTG

GCGCTCATGTCCACGGGCAGCCTGCACCAGCTCCACATATTCATCTTCGT

GCTAGCCGTCTTCCATGTCACCTACAGCGTCATCATCATGGCTCTAAGCC

GTCTCAAGgtgagcctttctttctttctttcccgtgcttccagatcctgc

gcggttcccgggcaaggtggcgctcatcctacgtctgtctcagttaaacc

tgctaccaatccttaacctgctccggcatantattctnattcctnccccc

ggcagATGAGAACATGGAAGAAATGGGAGACAGAGACCGCCTCCTTGGAA

TACCAGTTCGCAAATggtcagacaattttccaaatgaaacctcttctgtt

ttgatgcatttacagaggcaggcatgatcagagcgagtgaactgatgata

tgttcttctctttcccgtgcttccaGATCCTGCGCGGTTCCGCTTCACGC

ACCAGACGTCGTTCGTGAAGCGGCACCTGGGCCTGTCCAGCACCCCCGGC

GTCAGATGGGTGGTGGCCTTCTTCAGGCAGTTCTTCAGGTCGGTCACCAA

GGTGGACTACCTCACCTTGAGGGCAGGCTTCATCAACgtacgtaataccc

caaaagccccctctccttctagctccgtcggccattgccgcgacgcttct

gaaataagtactgttccaacaccaatgatcacatgctctctctttccatg

attctgcgcagGCGCACTTGTCGCAGAACAGCAAGTTCGACTTCCACAAG

TACATCAAGAGGTCCATGGAGGACGACTTCAAAGTCGTCGTTGGCATcag

gtaggttgcattccatggatatgattatacaattgtcgtcaggctccata

tgatattgcttagcttccatatgatacaatactatcagtttgctgcgtca

tggtctttgcccctgctggtccttgttgcatgatcttgacacatttggcc

tcttttcgCAGCCTCCCGCTGTGGGCTGTGGCGATCCTCACCCTCTTCCT

TGATATCGACggtatggaccttgtctttgcccccttctctgttgccttgc

tgctaaaacacttgtaatttatttgtctcgtaaccaccgttcattttcta

acctttcccccctttctttctgctcataGGGATCGGCACACTCACCTGGG

TTTCTTTCATCCCTCTCATCgtaagtgcgaatttctccgccgaaagcaac

agccaaaccccatttgattgcaatgcgaaatcacacctaataataattca

aattgtcattgtccatctgtctttcccagATCCTCTTGTGTGTTGGAACC

AAGCTAGAGATGATCATCATGGAGATGGCCCTGGAGATCCAGGACCGGTC

GAGCGTCATCAAGGGGGCACCCGTGGTCGAGCCCAGCAACAAGTTCTTCT

GGTTCCACCGCCCCGACTGGGTCCTCTTCTTCATACACCTGACGCTGTTC

CAGAACGCGTTTCAGATGGCACATTTCGTGTGGACAGTggtacgccgcgg

atgaacttgtcagttaataatatgggtgtcaaggcaccaagtgctgctgc

tgatgaactgcactgacagagatttacctgtgtcgcaGGCCACGCCCGGC

TTGAAGGACTGCTTCCATATGAACATCGGGCTGAGCATCATGAAGGTCGT

GCTGGGGCTGGCTCTCCAGTTCCTGTGCAGCTACATCACCTTCCCCCTCT

ACGCGCTAGTCACAcaggtaataaaaccgttgatgaagatctctgaacaa

ttgctctgggagaggagaaacagcagccttaatcatctgtgtgcgctggc

tttgtacgCAGATGGGATCAAACATGAAGAGGTCCATCTTCGACGAGCAG

ACAGCCAAGGCGCTGACCAACTGGCGGAACACGGCCAAGGAGAAGAAGAA

GGTCCGAGACACGGACATGCTGATGGCGCAGATGATCGGCGACGCAACAC

CCAGCCGAGGCACGTCCCCGATGCCTAGCCGGGGCTCATCGCCGGTGCAC

CTGCTTCAGAAGGGCATGGGACGGTCTGACGATCCCCAGAGCGCACCGAC

CTCGCCAAGGACCATGGAGGAGGCTAGGGACATGTACCCGGTTGTGGTGG

CGCATCCTGTACACAGACTAAATCCTGCTGACAGGCGGAGGTCGGTCTCT

TCATCAGCCCTCGATGCCGACATCCCCAGCGCAGATTTTTCCTTCAGCCA

GGGATGAgacaagtttctgtattgatgttagtccaatgtatagccaacat

aggatgtgatgattcgtacaataagaaatacaattttttactgagtcaaa

aaatttaacctgctaagcacaaacaaaaaccctctcatctcaataaaaat

accaaatggcaatcagtctcataacataagaaagagctctgaagaaaa

>TaMlo-B1

ATGGCGGACGACGACGAGTACCCCCCAGCGAGGACGCTGCCGGAGACGCC

GTCCTGGGCGGTGGCCCTCGTCTTCGCCGTCATGATCATCGTGTCCGTCC

TCCTGGAGCACGCGCTCCATAAGCTCGGCCATgtaagttccttcccggaa

aaagtaaatgagtgtctgccccagtcagactcagctcatggcttcctcct

tgttggcgtgtgtaagcagTGGTTCCACAAGCGGCACAAGAACGCGCTGG

CGGAGGCGCTGGAGAAGATCAAGGCGGAGCTCATGCTGGTGGGCTTCATC

TCGCTGCTGCTCGCCGTGACGCAGGACCCCATCTCCGGGATATGCATCTC

CGAGAAGGCCGCCAGCATCATGCGGCCCTGCAAGCTGCCCCCTGGCTCCG

TCAAGAGCAAGTACAAAGACTACTACTGCGCCAAAcaggtgagcctgtcg

gagccggaacaaaaacacaaacaaattccggccggccggagtttcttctt

gttgttttcttcctgattggcttggcctaactggtgtggcgcgtgctttt

ctggCAGGGCAAGGTGTCGCTCATGTCCACGGGCAGCTTGCACCAGCTGC

ACATATTCATCTTCGTGCTCGCCGTCTTCCATGTCACCTACAGCGTCATC

ATCATGGCTCTAAGCCGTCTCAAAgtgagcctttctttctttctttcttt

cttttttaccgcgcgtctgtcctcggtaaaactgctaccacccatcctta

acctgccccggcgtactcttctttcctgctgcagATGAGAACCTGGAAGA

AATGGGAGACAGAGACCGCCTCCCTGGAATACCAGTTCGCAAATggtcag

acaatttccgaaatggaacctgattgatgcatttacaaacgcacgcaggc

aggcacgatcagaggagtgaactgatgacatgttttctctctctttcccg

tgcttccaGATCCTGCGCGGTTCCGCTTCACGCACCAGACGTCGTTCGTG

AAGCGGCACCTGGGCCTCTCCAGCACCCCCGGCGTCAGATGGGTGGTGGC

CTTCTTCAGGCAGTTCTTCAGGTCGGTCACCAAGGTGGACTACCTCACCT

TGAGGGCAGGCTTCATCAACgtacgtacmaaaamarmyccctccttctag

ctccgtcagccattgccgcgacacttctgaaataagtatatttccgacac

caatgatctcatgtcttctctttccacgattccgcgcagGCGCATTTGTC

GCATAACAGCAAGTTCGACTTCCACAAGTACATCAAGAGGTCCATGGAGG

ACGACTTCAAAGTCGTCGTTGGCATcaggtagcttacaacattccatgga

tacgactatacaattgccgccaggctccatatgatattgcttaagttcca

catgatacaatactatcgtttgctgcgtcatggtctttgctcctgctggt

cttccttgcgtgatcttgacacatttggcctcttttcgCAGCCTCCCGCT

GTGGTGTGTGGCGATCCTCACCCTCTTCCTTGACATTGACggtatggacc

ttgctaaaacacttgtaatttgtctcgtaaccaccgttcattttctaacc

ttcctttccccttctttctgctggcaGGGATCGGCACGCTCACCTGGATT

TCTTTCATCCCTCTCGTCgtaagtgcgaatttctccgtcgaaagcaacag

ccagccccatttgattgcaatgcgaaaccacaccttaattgaaaatgtca

ttgtctgccttgtctttctcagATCCTCTTGTGTGTTGGAACCAAGCTGG

AGATGATCATCATGGAGATGGCCCTGGAGATCCAGGACCGGGCGAGCGTC

ATCAAGGGGGCGCCCGTGGTTGAGCCCAGCAACAAGTTCTTCTGGTTCCA

CCGCCCCGACTGGGTCCTCTTCTTCATACACCTGACGCTATTCCAGAACG

CGTTTCAGATGGCACATTTCGTGTGGACAGTggtatgtaccagtaattgg

cagttcagttagggatgcaaggcaccaagtagtgctgatgaactgcactg

acggagatttacttgttcgtaGGCCACGCCCGGCTTGAAGAAATGCTTCC

ATATGCACATCGGGCTGAGCATCATGAAGGTCGTGCTGGGGCTGGCTCTT

CAGTTCCTCTGCAGCTATATCACCTTCCCGCTCTACGCGCTCGTCACAca

ggtaataaagccgttgatgaagatgtctgaacaattgctctgggagagga

gtaacagcagccttaatcatgtaatctgtgtgatgggttgCAGATGGGAT

CAAACATGAAGAGGTCCATCTTCGACGAGCAGACGGCCAAGGCGCTGACA

AACTGGCGGAACACGGCCAAGGAGAAGAAGAAGGTCCGAGACACGGACAT

GCTGATGGCGCAGATGATCGGCGACGCGACGCCCAGCCGAGGGGCGTCGC

CCATGCCTAGCCGGGGCTCGTCGCCAGTGCACCTGCTTCACAAGGGCATG

GGACGGTCCGACGATCCCCAGAGCACGCCAACCTCGCCAAGGGCCATGGA

GGAGGCTAGGGACATGTACCCGGTTGTGGTGGCGCATCCAGTGCACAGAC

TAAATCCTGCTGACAGGAGAAGGTCGGTCTCGTCGTCGGCACTCGATGTC

GACATTCCCAGCGCAGATTTTTCCTTCAGCCAGGGATGAgacaagtttct

gtattgatgttagtccaatgtatagccaacataggatgtcatgattcgta

caataagaaatacaaatttttactgagtcaaaaaatttaacctgctaagc

acaaacaaaaaccctctcatctctaaaaaataccaaatggcaatcagtct

cataacataagaaagagctctgaagaaaa

>TaMlo-D1

ATGGCGGAGGACTACGAGTACCCCCCGGCGCGGACGCTGCCGGAGACGCC

GTCCTGGGCGGTGGCGCTCGTCTTCGCCGTCATGATCATCGTGTCCGTCC

TCCTGGAGCACGCGCTCCACAAGCTCGGCCATgtaagttccctcactcct

gcaacaagaaaaaaaaaagcctcaaccagaatcagcagctcagctcatgg

cttcctctgctcccttggtgcacctgcagTGGTTCCACAAGCGGCACAAG

AACGCGCTGGCGGAGGCGCTGGAGAAGATCAAAGCGGAGCTGATGCTGGT

GGGGTTCATCTCGCTGCTGCTCGCCGTGACGCAGGACCCAATCTCCGGGA

TATGCATCTCCGAGAAGGCCGCCAGCATCATGCGGCCCTGCAGCCTGCCC

CCTGGTTCCGTCAAGAGCAAGTACAAAGACTACTACTGCGCCAAAaaggt

gagcctgctacaagctactcccggagacggccgggaaaaacacaaacaga

ttccggcggccggccggagtttcttcttgtttccttcctgattggcttgg

cctaattggtgtgtgtttttctggAAGGGCAAGGTGTCGCTAATGTCCAC

GGGCAGCTTGCACCAGCTCCACATATTCATCTTCGTGCTCGCCGTCTTCC

ATGTCACCTACAGCGTCATCATCATGGCTCTAAGCCGTCTCAAAgtgagt

ctgtcaggcctacctgttcatgcttcggtaaagcaataaaactacttgct

accaatccctaatctgctccctcaggcataatattgttccttctttcctg

ctgcagATGAGGACATGGAAGAAATGGGAGACAGAGACCGCCTCCTTGGA

ATACCAGTTCGCAAATggtcagacaatttccgaaatgaaacctgactgat

gcatttacaaacgcacgcaggcaggcatgatcagagtgagtgaactgatg

atatgttttctctctctttcccgtgcctccaGATCCTGCGCGGTTCCGCT

TCACGCACCAGACGTCGTTCGTGAAGCGTCACCTGGGCCTCTCCAGCACC

CCCGGCATCAGATGGGTGGTGGCCTTCTTCAGGCAGTTCTTCAGGTCGGT

CACCAAGGTGGACTACCTCACCCTGAGGGCAGGCTTCATCAACgtacgta

ccaaaacaaatcctctccctctagcttcgccattgctgcgacgcttctga

aatatgtaccgttccgacaccagcgatctcatgtcttctctttccacgat

tctgcgcagGCGCATTTGTCGCATAACAGCAAGTTCGACTTCCACAAGTA

CATCAAGAGGTCCATGGAGGACGACTTCAAAGTCGTCGTTGGCATcaggt

aggttacattccatggataggattataaaattgccgtcaggctccatatg

atattgcttaggttccacatgatacaatactatcagtttgctgcgtcatg

gtctttgcccctgctggtcttccttgcgtgatcttgacacatttggcctc

ttttcgCAGCCTCCCGCTGTGGTGTGTGGCGATCCTCACCCTCTTCCTTG

ATATTGACggtatggaccttgctaaaacacttgtaatttgtctcgtaacc

accgttcattttctaaccttcctttccccttctttctgctggcaGGGATC

GGCACGCTCACCTGGATTTCTTTCATCCCTCTCGTCgtaagtgcgaattt

ctccgccgaaagcaacagccagccccatttgattgcaatgcgaaaccaca

ccttaattgaaaatgtcattgtctgtcttgtctttctcagATCCTCTTGT

GTGTTGGAACCAAGCTGGAGATGATCATCATGGAGATGGCCCTGGAGATC

CAGGACCGGGCGAGCGTCATCAAGGGGGCGCCCGTGGTTGAGCCCAGCAA

CAAGTTCTTCTGGTTCCACCGCCCCGACTGGGTCCTCTTCTTCATACACC

TGACGCTGTTCCAGAATGCGTTTCAGATGGCACATTTCGTCTGGACAGTg

gtatgtaccagtaattggcagttcggttagggatgcaaggcaccacgtgc

tgctgctgatgaactggactgacggagatttacttgtgtcgcaGGCCACG

CCCGGCTTGAAGAAATGCTTCCATATGCACATCGGTCTGAGCATCATGAA

GGTCGTGCTGGGGCTGGCTCTTCAGTTCCTCTGCAGCTATATCACCTTCC

CCCTCTACGCGCTCGTCACAcaggtaataaagccgttgatgaagatgtct

gaacaattgctctgggagaggagtaacagcagccttaatcatgtaatctg

tgtgatgggttgCAGATGGGATCGAACATGAAGAGGTCCATCTTCGACGA

GCAGACGGCCAAGGCGCTGACCAACTGGCGGAACACGGCCAAGGAGAAGA

AGAAGGTCCGAGACACGGACATGCTGATGGCGCAGATGATCGGCGACGCG

ACGCCCAGCCGAGGCACGTCGCCGATGCCTAGCCGGGCTTCGTCACCGGT

GCACCTGCTTCACAAGGGCATGGGACGGTCCGACGATCCCCAGAGCGCGC

CGACCTCGCCAAGGACCATGGAGGAGGCTAGGGACATGTACCCGGTTGTG

GTGGCGCATCCCGTGCACAGACTAAATCCTGCTGACAGGCGGAGGTCGGT

CTCTTCGTCGGCACTCGATGCCGACATCCCCAGCGCAGATTTTTCCTTCA

GCCAGGGATGAgacaagtttatgtattgatgttagtccaatgtatagcca

acataggatgtcatgattcgtacaataagaaatacaaatttttactgagt

caaaaatttaacctgctaagcacaaacaaaaaccctctcatctatgtaaa

aataccaaatggcaatcagtctcataacataagaaatagctctgaagaaa
