# Supplementary material for: mlo‐based powdery mildew resistance in hexaploid bread wheat generated by a non‐transgenic TILLING approach
Source: Plant Biotechnol J. 2016 Sep 25;15(3):367–78. doi: 10.1111/pbi.12631 (PMC5316926; doi:10.1111/pbi.12631)
Supplement: Supplementary file 11 — File S3 Supplementary Materials and Methods. [file PBI-15-367-s006.docx]

**File S3** Supplementary Materials and Methods

**Plant growth**

Barley and wheat seedlings were sown in soil composed of natural clay, peat, and perlite, type VM (Einheitserde, Sinntal-Altengronau, Germany) and grown under controlled conditions (local walk-in chamber equipped with metal-halide lamps or Panasonic MLR-352 plant growth cabinet (Ewald Innovationstechnik GmbH, Bad Nenndorf, Germany) equipped with fluorescent tubes), at 20°C and a 16 h photoperiod with a photosynthetic photon flux density (PPFD) of ca. 80-100 µmol m^-2^ s^-1^ and ambient relative humidity. For a complete growth cycle, seedlings were transferred to soil with a mixture of natural clay and peat, type ED 73 (Einheitserde, Sinntal-Altengronau, Germany), and grown in controlled conditions as described above or in the greenhouse with supplemental light (metal-halide lamps) if necessary. Wheat seeds from the TILLING population were first surface-sterilized as follows: grains were washed with 70% ethanol (Carl Roth GmbH, Karlsruhe, Germany) for 5 min with gently shaking, then washed with 10% hypochlorite solution plus one drop of Tween 20 (Sigma, Deisenhofen, Germany) with gently shaking, and finally rinsed thoroughly with sterile water. Seeds were then transferred to petri dishes containing damp sterile filter paper and maintained for the first week at 12°C in darkness. Afterwards, germinated seeds were moved to pots with soil as mentioned above. Winter wheat seeds were surface sterilized, sown in soil, and kept under controlled growth conditions (see above) for the first 10 days. Thereafter they were moved to 4°C, 16 h photoperiod for 20 days (vernalization) and finally transferred back to controlled growth conditions as mentioned above.

**Pathogen assays**

For pathogen assays, the first leaf of seedlings of 10 day-old (wheat) or 7 day-old (barley) plants were fixed with surgical tape to a polycarbonate platform and inoculated with *Bgt* (wheat) or *Bgh* (barley) conidiospores. Leaves were collected at 72 hpi (wheat) or 48 hpi (barley), fixed and cleared in a solution containing 2:1 ethanol:stock solution (stock solution: 1:2:1 (lactic acid:ethanol:ddH_2_O)).

Epiphytic fungal structures were stained with Coomassie Brillant Blue R-250 (Carl Roth GmbH, Karlsruhe, Germany). The host cell entry rate was calculated as: (number of attacked cells with haustoria and or hyphae/ attacked cells)*100 on the basis of 200 interaction sites per leaf. Only cells attacked by one spore were evaluated. Stomata, trichomes, and cells lying above vascular bundles were excluded from the assessment. Three to four leaves were evaluated per mutant/genotype in one experiment and at least three independent biological replicates were performed. All pathogen assays were performed under the plant growth conditions described above (see section “Plant growth”).

**Identification of intron sequences of *TaMlo-A1*, *TaMlo-B1* and *TaMlo-D1***

Several NCBI accessions were used to complete the genomic sequences of the *TaMlo* homoeologues. 1) *TaMlo-A1*: AF361933 and AX063298; 2) *TaMlo-B1*: AF361932, AX063294, AF384145; and 3) *TaMlo-D1*: AX063296. Primers TaMlo- 1aF, 1bF, 1R, 2F and 2F (Table S1) were designed based on the exon sequences to identify missing introns. Additional genomic sequences from cv. Chinese Spring were generated from a combination of IWGSC genome survey data (IWGSC, 2014) obtained from wheat-urgi.versailles.inra.fr and shotgun (454 pyrosequencing) sequences from cerealsdb.uk.net (Brenchley *et al.*, 2012).

**Primer design for TILLING**

Alignment of the sequences was performed by Unipro UGENE (Okonechnikov *et al.*, 2012). Homoeologue-specific primers for TILLING (Table S1) were manually designed, inspected by FastPCR (Kalendar *et al.*, 2009) and validated by PCR using genomic DNA from cv. Cadenza and sequencing. Genomic DNA was isolated from 10 day-old wheat leaves using the urea-phenol method (Shure *et al.*, 1983).

**Cloning of *TaMlo* homoeologues**

Extraction of RNA from 8 day-old leaves from cv. Cadenza was performed with the RNeasy Mini Kit (Quiagen GmbH, Hilden, Germany). For cDNA synthesis, SuperScript II^TM^ reverse transcriptase and an oligo (dT) 18-mer primer (Invitrogen, Life Technologies GmbH, Darmstadt, Germany) were used.

*TaMlo-A1*: Primer pair TaMlo_A1_PGWY_F/TaMlo_ALL_PGWY_R (Table S1) was used for a 1^st^ round PCR. This product was used as template for a 2^nd^ round PCR with primer pair TaMlo_A1_GWY_F ­­/TaMlo_ALL_GWY_R (Table S1). The amplification product was purified from the agarose gel with the NuceoSpin Gel and PCR clean-up kit (Macherey-Nagel, Düren, Germany) and used for cloning with Gateway^®^ technology (Invitrogen, Life Technologies GmbH, Darmstadt, Germany). Gateway^®^ BP and LR recombination reactions were performed with the vectors pDONR201^TM^ (Invitrogen, Life Technologies GmbH, Darmstadt, Germany) and pUbiGATE (maize *Uiquitin1* promoter::attR-cassette::nos-polyA signal, Gateway^®^-compatible overexpression vector; Miklis, 2004), respectively. Confirmation by DNA sequencing was carried out with pDONR201- and pUbiGATE-specific primers (Table S1). Competent *Escherichia coli* DH5α^®^ cells were used for transformation.

*TaMlo-B1 and TaMlo-D1:* Primer pairs TaMlo_B1_TOPO_F/TaMlo_ALL_TOPO_R and TaMlo_D1_TOPO_F/TaMlo_ALL_TOPO_R were used for PCR (Table S1). The amplification products were cloned into vector pCR-BluntII-TOPO^®^ using the Zero Blunt^®^ TOPO^®^ kit (Invitrogen, Life Technologies GmbH, Darmstadt, Germany). TOPO^®^ plasmids were used as template for cloning with Gateway^®^ technology (Invitrogen, Life Technologies GmbH, Darmstadt, Germany) (Invitrogen) using the primer pairs TaMlo_B1_GWY_F/TaMlo_ALL_GWY and TaMlo_D1_GWY_F /TaMlo_ALL_GWY_R (Table S1). For Gateway^®^ BP and LR recombination reactions the same vectors and competent cells as for *TaMlo-A1* were employed.

**Site-directed mutagenesis of *TaMlo* and barley *Mlo***

Mutations were introduced into the respective *TaMlo* WT coding sequence *via* PCR-splicing by overlap extension (Horton *et al.*, 1989) as described previously in Reinstädler *et al.* (2010). Primers for different mutations are given in Table S1.

WT barley *Mlo* has been cloned previously (Reinstädler *et al.*, 2010) and was used as a template to generate the mutant versions *mlo* (P324A) and *mlo*-38 (primers in Table S1). To generate the mutant *mlo*-29, the existing clone pAM-PAT-mlo29 was used as a template to amplify the gene for the Gateway^®^ system (primers Mlo-GWY-F and Mlo_GWY_R), following the same protocol as described for *TaMlo-A1*.

**Prediction of functionality of *Ta*Mlo mutant versions**

We predicted the effect of amino acid substitutions on *Ta*Mlo function with the online tool SIFT (Kumar *et al.*, 2009; <http://sift.jcvi.org/>). The analysis was performed for each *Ta*Mlo homoeologue individually with the single protein option “SIFT sequence” (<http://sift.jcvi.org/www/SIFT_seq_submit2.html>), which involves a PSI-BLAST search using the UniRef90 database (April 2011 version), setting median conservation of sequences = 1.00 and removing sequences with more than 75% identity to the query.

**Statistical Analysis**

We used a generalized linear model (GLM) to perform the statistical analysis for Figures 2 and 3 since the data from microscopic evaluation of powdery mildew infection, given as host cell entry, (1) expresses a rate, (2) is considered proportion data, (3) has a small number of biological repetitions (n= at least 3) and (4) assumes an unequal variance and non-normal distribution (Crawley, 2015). We employed GLM with family=binomial, and due to overdispersion we used quasibinomial errors to fit the model. The single, double and triple asterisks above the boxplots indicate differences that are statistically highly significant (****P*<0.001), very significant (***P*<0.01) and significant (**P*<0.5) respectively. For Figures 4a and S2a, additional statistical analysis was conducted on the basis of the Games-Howell *post-hoc* test for pair-wise testing of all groups (genotypes). The tests were performed with the software R, version 3.2.2 (R Core Team, 2013) and the box plots were generated using the open-source application BoxPlotR (Spitzer *et al.*, 2014). The R script for the Games-Howell *post-hoc* test can be found in File S4.

**CAPS markers for genotyping**

We used the free web tools from NEBCutter (Vincze *et al.*, 2003) to analyze the sequences of the 2^nd^ round PCR products to find suitable restriction enzymes to discriminate the WT form from the mutant variants. Restriction reactions were performed as advised by the manufacturer (normally overnight) and products run in 2% agarose gel, containing ethidium bromide for visualization.

**Transcript accumulation of the *TaMlo* homoeologues**

Mapping of the paired-end RNA-seq reads to the wheat reference transcriptome containing the coding sequences of *TaMlo* obtained in this study (File S1) was performed as described previously in Pearce *et al.* (2015).

**Cloning of short fragments for *TaMlo* expression analysis**

RNA extraction and cDNA synthesis were performed as described above in the section “Cloning of *TaMlo* homoeologues”. Primers TaMlo-ABD-F and TaMlo-ABD-R (Table S1) were used for amplification of the fragment. The PCR product was cloned into vector pCR-BluntII-TOPO^®^ using the Zero Blunt^®^ TOPO^®^ kit (Invitrogen, Life Technologies GmbH, Darmstadt, Germany). Clones were sequenced using the common primer M13R (Table S1) and sequence alignment was performed using the software Unipro UGENE (Okonechnikov *et al.*, 2012).

**References for Supplementary Materials and Methods**

Brenchley, R., Spannagl, M., Pfeifer, M., Barker, G.L.A., D’Amore, R., Allen, A.M., *et al.* (2012) Analysis of the bread wheat genome using whole-genome shotgun sequencing. *Nature*, **491**, 705–710.

Crawley, M. (2015) Statistics: an introduction using R. 2nd edn. John Wiley & Sons, Ltd.

Horton, R.M., Hunt, H.D., Ho, S.N., Pullen, J.K. and Pease, L.R. (1989) Engineering hybrid genes without the use of restriction enzymes: gene splicing by overlap extension. *Gene*, **77**, 61–68.

Kalendar, R., Lee, D. and Schulman, A.H. (2009) FastPCR software for PCR primer and probe design and repeat search. *Genes, Genomes and Genomics*, **3**, 1–14.

Kumar P., Henikoff S. and Ng P.C. (2009) Predicting the effects of coding non-synonymous variants on protein function using the SIFT algorithm. *Nat. Protoc.* 4, 1073-1081.

Miklis, M. (2004) A high-throughput procedure for the identification of genes contributing to plant defence mechanisms. PhD thesis, Universitat zu Köln. Available online at http://kups.ub.uni-koeln.de/1420/

Okonechnikov, K., Golosova, O., Fursov, M., Varlamov, A., Vaskin, Y., Efremov, I., *et al.* (2012) Unipro UGENE: A unified bioinformatics toolkit. *Bioinformatics*, **28**, 1166–1167.

Pearce, S., Huttly, A.K., Prosser, I.M., Li, Y.-D., Vaughan, S.P., Gallova, B., *et al.* (2015) Heterologous expression and transcript analysis of gibberellin biosynthetic genes of grasses reveals novel functionality in the GA3ox family. *BMC Plant Biol.* **15**, 130.

R Core Team. (2013) R: A language and environment for statistical computing. Vienna, Austria: The R foundation for statistical computing. ISBN: 3-900051-07-0. Available online at <http://www.R-project.org/>.

Reinstädler, A., Müller, J., Czembor, J.H., Piffanelli, P. and Panstruga, R. (2010) Novel induced *mlo* mutant alleles in combination with site-directed mutagenesis reveal functionally important domains in the heptahelical barley Mlo protein. *BMC Plant Biol.* **10**, 31.

Shure, M., Wessler, S. and Fedoroff, N. (1983) Molecular identification and isolation of the *Waxy* locus in maize. *Cell*, **35**, 225–233.

Spitzer, M., Wildenhain, J., Rappsilber, J. and Tyers, M. (2014) BoxPlotR: a web tool for generation of box plots. *Nat. Methods*, **11**, 121–2.

Vincze, T., Posfai, J. and Roberts, R.J. (2003) NEBcutter: A program to cleave DNA with restriction enzymes. *Nucleic Acids Res.* **31**, 3688–3691.
