# Supplementary material for: mlo‐based powdery mildew resistance in hexaploid bread wheat generated by a non‐transgenic TILLING approach
Source: Plant Biotechnol J. 2016 Sep 25;15(3):367–78. doi: 10.1111/pbi.12631 (PMC5316926; doi:10.1111/pbi.12631)
Supplement: Supplementary file 12 — File S4 R script for the Games–Howell post hoc test. [file PBI-15-367-s010.docx]

**File S4** R script for the Games-Howell *post-hoc* test.

#post-hoc test

tukey <- function(data,group,method=c("Tukey", "Games-Howell")) {

OK <- complete.cases(data, group)

data <- data[OK]

group <- factor(group[OK])

n <- tapply(data, group, length)

a <- length(n)

phi.e <- sum(n)-a

Mean <- tapply(data, group, mean)

Variance <- tapply(data, group, var)

result1 <- cbind(n, Mean, Variance)

rownames(result1) <- paste("Group", 1:a, sep="")

method <- match.arg(method)

if (method == "Tukey") {

v.e <- sum((n-1)*Variance)/phi.e

t <- combn(a, 2, function(ij)

abs(diff(Mean[ij]))/sqrt(v.e*sum(1/n[ij])) )

p <- ptukey(t*sqrt(2), a, phi.e, lower.tail=FALSE)

Tukey <- cbind(t, p)

rownames(Tukey) <- combn(a, 2, paste, collapse=":")

return(list(result1=result1, Tukey=Tukey, phi=phi.e, v=v.e))

}

else {

t.df <- combn(a, 2, function(ij) {

t <- abs(diff(Mean[ij]))/sqrt(sum(Variance[ij]/n[ij]))

df <- sum(Variance[ij]/n[ij])^2/sum((Variance[ij]/n[ij])^2/(n[ij]-1))

return(c(t, df))} )

t <- t.df[1,]

df <- t.df[2,]

p <- ptukey(t*sqrt(2), a, df, lower.tail=FALSE)

Games.Howell <- cbind(t, df, p)

rownames(Games.Howell) <- combn(a, 2, paste, collapse=":")

return(list(result1=result1, Games.Howell=Games.Howell))

}

}

tukey1<-tukey(value,genotype)

summary(tukey1)

tukey1
